# A nonlinear windkessel model for cardiovascular dynamics: Variable compliance and pathological simulations

**DOI:** 10.1007/s11517-026-03638-5

**Published:** 2026-07-27

**Authors:** Marcus Varanis, Mateus Ferraz, Daniel Longo, José Manoel Balthazar

**Affiliations:** 1 Nonlinear Dynamics and Signal Processing Group - Institute of Physics, Federal University of Mato Grosso do Sul, Bloco V - R. Ufms, Campo Grande, 79070900 MS Brazil; 2 Faculty of Mechanical Engineering, Universidade Estadual Paulista, Av. Eng. Luis Edmundo Carrijo Coube, 2681, Bauru, 17033360 SP Brazil

**Keywords:** Cardiovascular modeling, Closed-loop Windkessel model, Pressure-dependent aortic compliance, Arterial stiffness, Hemodynamic simulation

## Abstract

**Abstract:**

Lumped-parameter cardiovascular models are computationally efficient and physiologically interpretable, but many still represent arterial compliance as linear and pressure-independent. Here, we introduce a nonlinear extension of a classical closed-loop Windkessel model by defining aortic compliance as an exponential function of aortic pressure. The heart, systemic circulation, and pulmonary circulation are represented as an RLC network with time-varying ventricular elastances, yielding a twelve-state formulation derived from Kirchhoff’s laws. Under nominal conditions, the nonlinear model recovers the linear baseline, with near-superimposable pressure and flow waveforms and physiologically plausible steady-state indices. Away from the nominal operating point, the pressure-dependent law selectively modulates proximal pulsatility, while mean arterial pressure, stroke volume, and cardiac output remain comparatively preserved. Targeted parameter changes produce qualitative waveform and index-level signatures consistent with aortic regurgitation and arterial stiffening. The proposed formulation provides a compact, physiologically grounded platform for sensitivity analysis, pathological emulation, and early-stage evaluation of cardiovascular modeling scenarios.

**Graphical abstract:**

**Figure:**
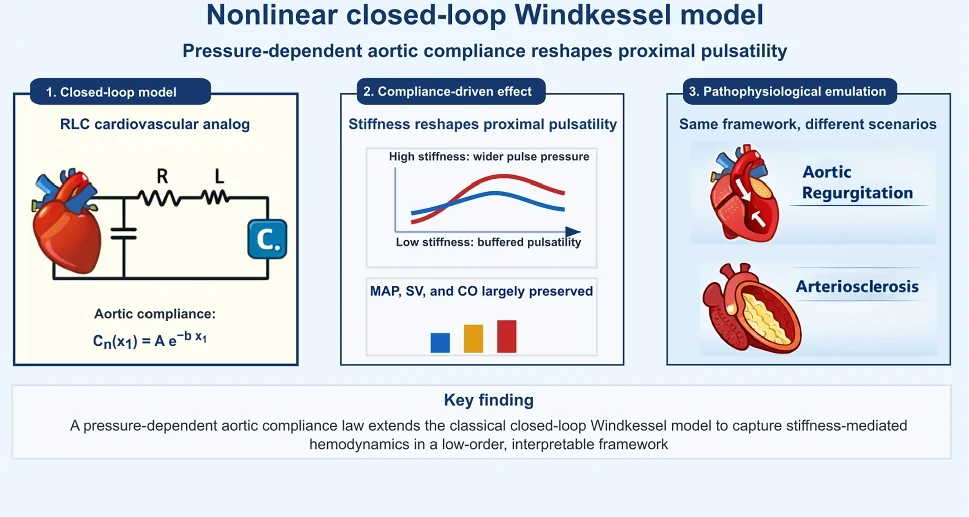

## Introduction

The cardiovascular system operates across multiple spatial and temporal scales. At the organ level, the heart drives blood through a branching vascular network; at smaller scales, inertial, viscous, and elastic effects shape the propagation and damping of pulsatile waves. Mathematical modeling provides a mechanistic framework for interpreting these interactions and for exploring scenarios that are impractical or unethical to study *in vivo* [1, 2].

Zero-dimensional, or lumped-parameter, formulations represent the heart and vasculature as interconnected compartments linked by resistive, capacitive, and inductive elements. These models are computationally inexpensive and provide access to global hemodynamic indices, including pressure-volume loops and cardiac output. They have therefore been used for sensitivity analysis, inverse parameter estimation, and early-stage evaluation of cardiovascular devices [3, 4]. One-dimensional and two-dimensional models enrich this description by resolving wave propagation and reflections along compliant tubes and networks [5–7]. Fully three-dimensional models capture local flow patterns and wall deformation but require detailed anatomical data and substantial computational resources [8]. The choice of model dimensionality, therefore, depends on the balance between physiological detail and computational feasibility required by each application.

Within the 0D family, the Windkessel concept has become a cornerstone. Originally proposed by Frank and later extended by Westerhof and co-workers, the Windkessel analog formalizes the correspondence between vascular hemodynamics and electrical RLC networks [9, 10]. Classical three-element and four-element Windkessel models assume constant arterial compliance and have been extended with inertial and resistive elements, time-varying ventricular elastance, and valve dynamics [11–13]. Closed-loop formulations couple the left and right heart chambers to the systemic and pulmonary vascular beds, enabling clinically interpretable waveforms and supporting simulations of valvular dysfunction and atrioventricular interaction [14, 15].

A persistent limitation of many lumped-parameter models is the representation of large-artery compliance as a linear, pressure-independent parameter. Experimental studies show that arterial walls exhibit nonlinear stress-strain behavior and that effective stiffness increases with pressure and age. Constant linear compliance can therefore underrepresent pressure-dependent changes in systolic loading and pulse pressure under hypertensive, aging, or disease-related conditions. Several authors have proposed nonlinear or viscoelastic extensions of the Windkessel model, including pressure-dependent compliance elements and fractional-order representations [16–22]. Recent studies have revisited these ideas within modern closed-loop frameworks and multiscale couplings [3, 4, 23, 24]. Capturing the essential nonlinearity of arterial compliance within a parsimonious and interpretable model remains a central challenge for reduced-order cardiovascular modeling.

We introduce a nonlinear extension of a classical closed-loop Windkessel model by expressing aortic compliance as an exponential function of aortic pressure. The circulatory system is formulated as a twelve-state network of resistors, inertances, and compliant elements, with time-varying elastance describing ventricular contractility. Embedding the pressure-dependent compliance in the aortic branch preserves the linear baseline near nominal pressures while allowing effective stiffness to vary dynamically with the instantaneous aortic state. The formulation remains computationally lightweight and provides a controlled way to study stiffness-mediated changes in proximal pulsatility and pressure-volume dynamics. In benchmark comparisons, the nonlinear law selectively modulates pulse pressure while mean arterial pressure, stroke volume, and cardiac output remain comparatively preserved. Targeted parameter changes further reproduce qualitative waveform signatures consistent with aortic stiffening and aortic regurgitation.

Section 2 presents the physics-based closed-loop formulation, including ventricular dynamics, valve mechanisms, constitutive laws, numerical integration, and the baseline parameter set. Section 3 compares the nonlinear formulation with the linear baseline under nominal, perturbed, and pathological scenarios. Section 4 discusses the physiological interpretation, modeling implications, and limitations of the results, and Section 5 summarizes the main findings and outlines future extensions toward subject-specific calibration.

## Physics-based model for the cardiovascular system

**Fig. 1:**
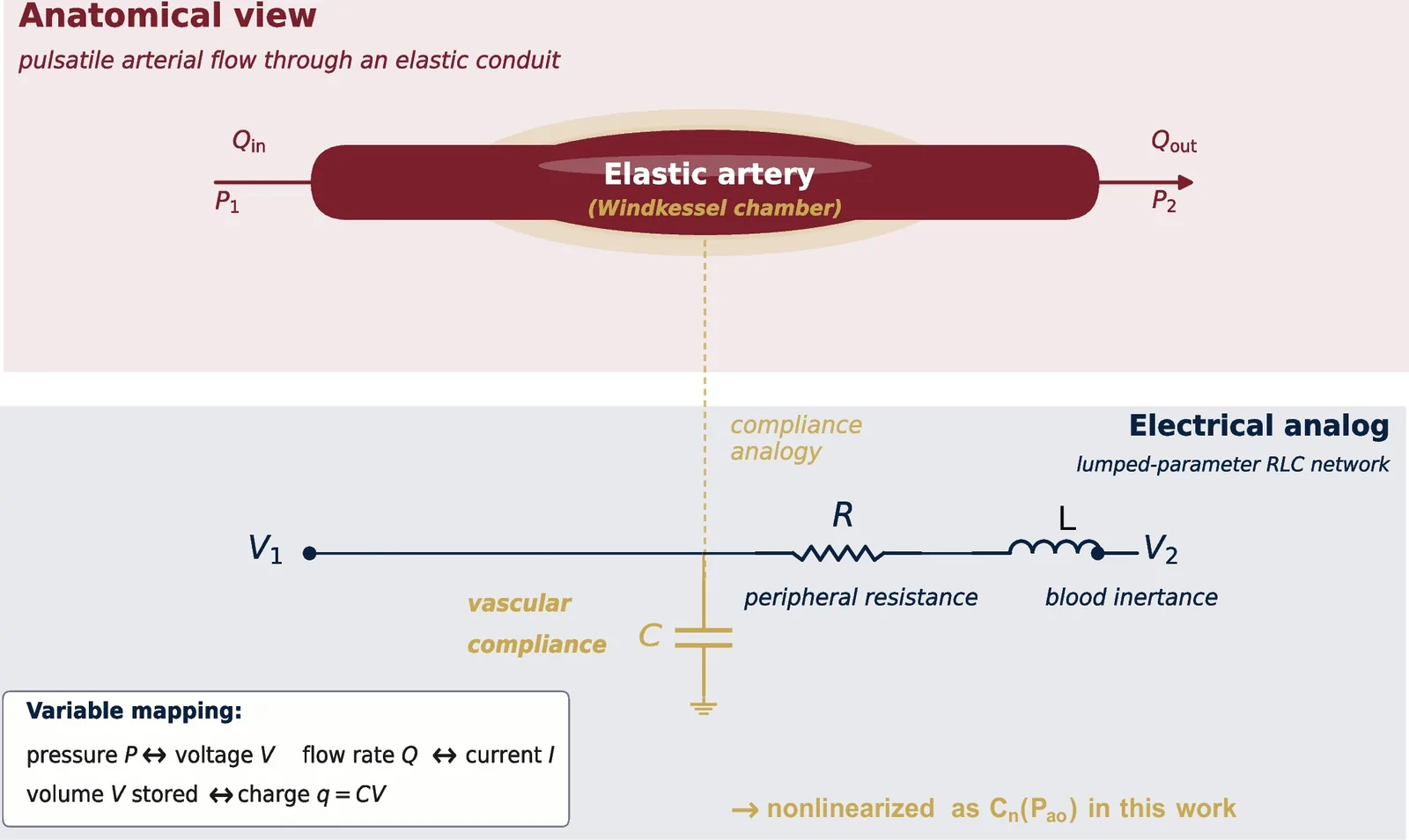
Schematic representation of the Windkessel effect and its fluid-electrical analog. A distensible arterial segment stores blood during systole and recoils during diastole, smoothing pulsatile inflow ($P_{1}$) into outflow ($P_{2}$). In the RLC representation, pressure maps to voltage, flow to current, and stored arterial volume to capacitor charge. The compliance *C* represents elastic storage and is generalized here as $C_{n}(P_{\textrm{ao}}) = A\, e^{-b\,P_{\textrm{ao}}}$

We formulate the cardiovascular system as a closed-loop, Windkessel-type electrical analog. In this framework, the dominant mechanisms of macrocirculatory hemodynamics are represented by an RLC network: resistive elements account for viscous pressure losses, capacitive elements describe compliant volume storage in distensible compartments, and inductive elements represent blood inertance. The Windkessel buffering mechanism, by which compliant arteries store blood during systole and sustain flow during diastole, together with the fluid-electrical correspondence adopted in the model, is summarized in Fig. 1.

**Fig. 2:**
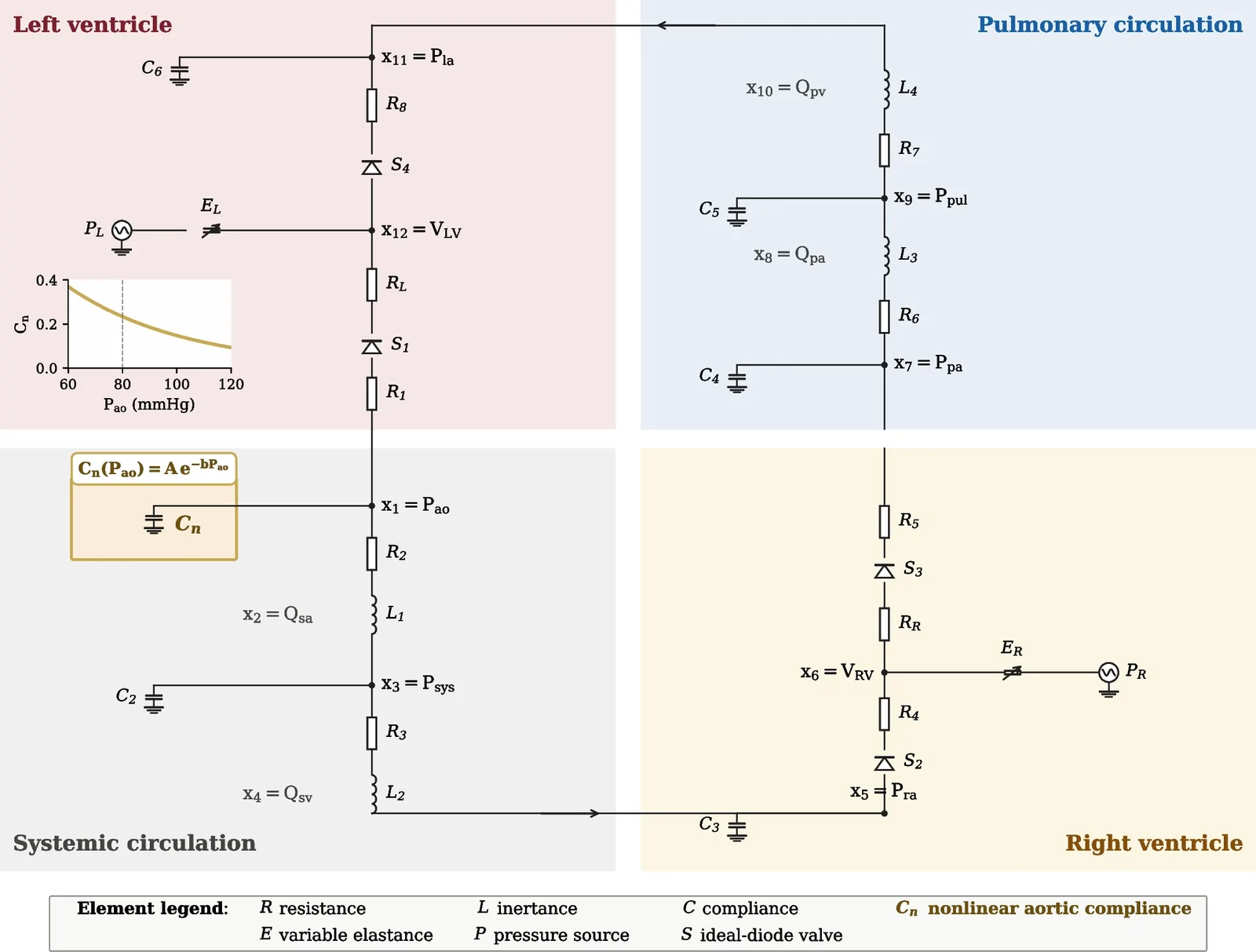
Closed-loop RLC analog of the twelve-state nonlinear cardiovascular model. Ideal-diode valves $S_{1}$ through $S_{4}$ connect the left ventricle, systemic circulation, right ventricle, and pulmonary circulation in an oriented closed loop. Ventricular pumping is modeled by the time-varying elastances $E_{L}(t)$ and $E_{R}(t)$ coupled to the pressure sources $P_{L}$ and $P_{R}$. The pressure-dependent aortic compliance $C_{n}(P_{\textrm{ao}})=A\,e^{-bP_{\textrm{ao}}}$ is the only nonlinear storage element; all other capacitive elements are linear. The inset compares this law with the linear baseline $C_{n}\equiv C_{n}(P_{0})$

The closed-loop architecture is shown in Fig. 2. The systemic and pulmonary circulations are coupled to the left and right heart through four chambers and unidirectional valves, forming an oriented cardiovascular loop. This decomposition yields a compact and physiologically interpretable framework in which local subsystem modifications can be introduced while preserving global consistency. It therefore supports parameter identification, mechanistic interpretation, and controlled perturbations for pathological or device-oriented simulations.

Consistent with the classical Windkessel analogy [9], each main physiological component is assigned an electrical counterpart. Ventricular pumping is represented by time-varying elastance branches coupled to myocardial viscous resistances, yielding pressure-generating units that drive the circulation. Valves are modeled as ideal diodes in series with resistances, enforcing unidirectional flow while retaining transvalvular dissipation. The atria act as compliant, low-pressure reservoirs. The systemic and pulmonary vascular beds are each described by two-stage RLC networks, where *R*, *L*, and *C* represent viscous dissipation, blood inertance, and elastic storage in vessel walls, respectively. Within this modeling class, arterial compliance is a primary determinant of waveform morphology and global hemodynamic buffering, as supported by extensive characterizations of arterial pressure-volume behavior [25–28].

The central modeling step is the replacement of constant aortic compliance by a nonlinear pressure-dependent element. This formulation relaxes a standard assumption of classical lumped-parameter cardiovascular models by allowing compliance to vary with the instantaneous aortic pressure. Arterial stiffening is therefore introduced in a low-order model through pressure-dependent changes in effective compliance, which primarily modulate proximal pulsatility and flow buffering within the closed-loop system. In Fig. 2, this nonlinearity is localized in the aortic compliance branch, enabling direct comparison between the linear and nonlinear formulations under otherwise identical architecture and parameterization.

The complete model is written as a system of ordinary differential equations obtained from Kirchhoff’s laws applied to the circuit topology. The dynamics are described by twelve state variables spanning pressures, flow rates, and ventricular volumes, each with a direct physiological interpretation and a corresponding electrical analog (Table 1).

**Table 1:** State variables of the twelve-state closed-loop cardiovascular model, with their corresponding electrical analogs and cardiovascular quantities

State variable	Electrical analog	Cardiovascular quantity
$x_1$	Voltage across $C_n$	Aortic pressure
$x_2$	Current through $L_1$	Systemic arterial blood flow
$x_3$	Voltage across $C_2$	Systemic pressure
$x_4$	Current through $L_2$	Systemic venous blood flow
$x_5$	Voltage across $C_3$	Right atrial pressure
$x_6$	Charge (elastance branch $E_R$)	Right ventricular volume
$x_7$	Voltage across $C_4$	Pulmonary arterial pressure
$x_8$	Current through $L_3$	Pulmonary arterial blood flow
$x_9$	Voltage across $C_5$	Pulmonary capillary pressure
$x_{10}$	Current through $L_4$	Pulmonary venous blood flow
$x_{11}$	Voltage across $C_6$	Left atrial pressure
$x_{12}$	Charge (elastance branch $E_L$)	Left ventricular volume

### RLC circuit analysis

The governing equations are obtained by combining the constitutive relations of the circuit elements with Kirchhoff’s laws. Pressures and flow rates are mapped to voltages and currents, respectively, so the standard RLC relations provide a compact route to the state-space formulation.

For a resistive element, the pressure drop follows Ohm’s law,

1 $$\begin{aligned} v(t) = R\,i(t), \end{aligned}$$

where *R* denotes the hydraulic resistance associated with viscous dissipation.

For a capacitive element,

2 $$\begin{aligned} q(t) = C\,v(t), \end{aligned}$$

and, since $q(t)=\int i(t)\,dt$, differentiation gives

3 $$\begin{aligned} i(t) = C\,\frac{dv(t)}{dt}. \end{aligned}$$

In hemodynamic terms, this branch represents compliant volume storage, relating pressure variations to flow exchange in distensible compartments.

For an inductive element,

4 $$\begin{aligned} v(t) = L\,\frac{di(t)}{dt}, \end{aligned}$$

where *L* denotes blood inertance, namely the pressure contribution required to accelerate or decelerate the moving fluid mass.

The global closed-loop dynamics is obtained by applying Kirchhoff’s current law (KCL) at the circuit nodes and Kirchhoff’s voltage law (KVL) along the independent loops. Eqs. (1) through (4) define the local constitutive behavior of the network, whereas KCL and KVL impose flow conservation and pressure balance. Their combination yields the coupled ODE system governing the time evolution of pressures, flow rates, and chamber volumes.

#### Circulatory network and ventricular modeling

**Fig. 3:**
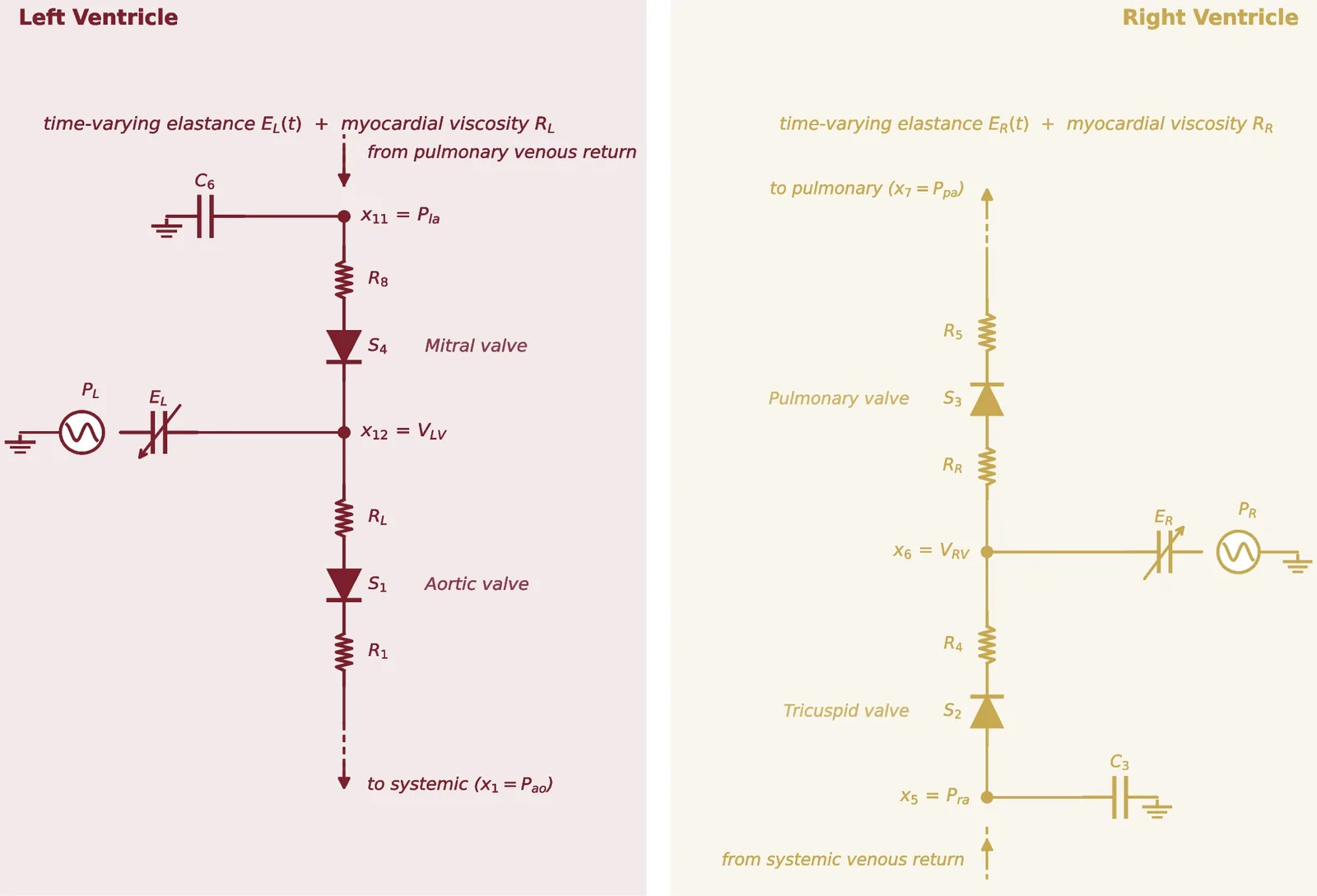
Electrical analogs of the left and right ventricles, isolated from the closed-loop model in Fig. 2. Each ventricle is modeled by a time-varying elastance, $E_{L}(t)$ or $E_{R}(t)$, in series with a pressure source, $P_{L}$ or $P_{R}$, and a myocardial viscous resistance, $R_{L}$ or $R_{R}$. Ideal-diode valves enforce unidirectional filling and ejection in both chambers. The ventricular volumes $x_{12}=V_{\textrm{LV}}$ and $x_{6}=V_{\textrm{RV}}$ are coupled to the elastance branches through the pressure-volume relation $P(t)=E(t)\bigl (V(t)-V_{\textrm{lp}}\bigr )$. Dashed boundary segments indicate coupling to the systemic and pulmonary circulations detailed in Fig. 4

Ventricular dynamics are described using a time-varying elastance formalism with a linearized pressure-volume-flow relation during systole, following Halaimi et al. [23]. Figure 3 shows the corresponding electrical analogs. Each ventricle is represented by an isovolumetric pressure source $P_i(t)$ in series with a time-dependent elastance *E(t)* and a myocardial viscous resistance $R_m$. This structure links ventricular pressure to the instantaneous chamber volume *V(t)* and its rate of change *dV/dt*, while preserving a compact representation suitable for closed-loop coupling.

The ventricular activation is prescribed by the function *a(t)*, which modulates isovolumetric pressure generation during each cardiac cycle:

5 $$\begin{aligned} a(t)= {\left\{ \begin{array}{ll} \dfrac{1-\cos \!\left( 2\pi \dfrac{t_m}{t_s}\right) }{2}, & t_m < t_s, \\ 0, & \text {otherwise}, \end{array}\right. } \end{aligned}$$

where $t_s$ is the systolic duration, defined as $t_s = 0.16 + 0.3\,t_c$, $t_c$ is the cardiac period, and $t_m={\text {mod}}(t,t_c)$ is the elapsed time within the current cardiac cycle.

With the diastolic elastance $E_d$ fixed, ventricular pressure is given by

6 $$\begin{aligned} P_v(t)= {\left\{ \begin{array}{ll} P(t) + E(t)\bigl (V(t)-V_{\textrm{lp}}\bigr ) + R_m\,\dfrac{dV}{dt}, & \text {systole}, \\ E_d\bigl (V(t)-V_{\textrm{lp}}\bigr ), & \text {diastole}, \end{array}\right. } \end{aligned}$$

where $V_{\textrm{lp}}$ is the linearization volume, $P(t)=P_0\,a(t)$ is the isovolumetric pressure input with peak value $P_0$, and $E(t)=E_d + E_s\,a(t)$ is the time-varying elastance. The coefficient $R_m$ represents viscous losses associated with myocardial contraction.

For the right and left ventricles, these relations become

7 $$\begin{aligned} \begin{aligned} P_R(t)&= P_{0R}\,a(t),&\qquad E_R(t)&= E_{dR}+E_{sR}\,a(t),\\ P_L(t)&= P_{0L}\,a(t),&\qquad E_L(t)&= E_{dL}+E_{sL}\,a(t), \end{aligned} \end{aligned}$$

where the subscripts *R* and *L* denote the right and left ventricles, respectively.

**Fig. 4:**
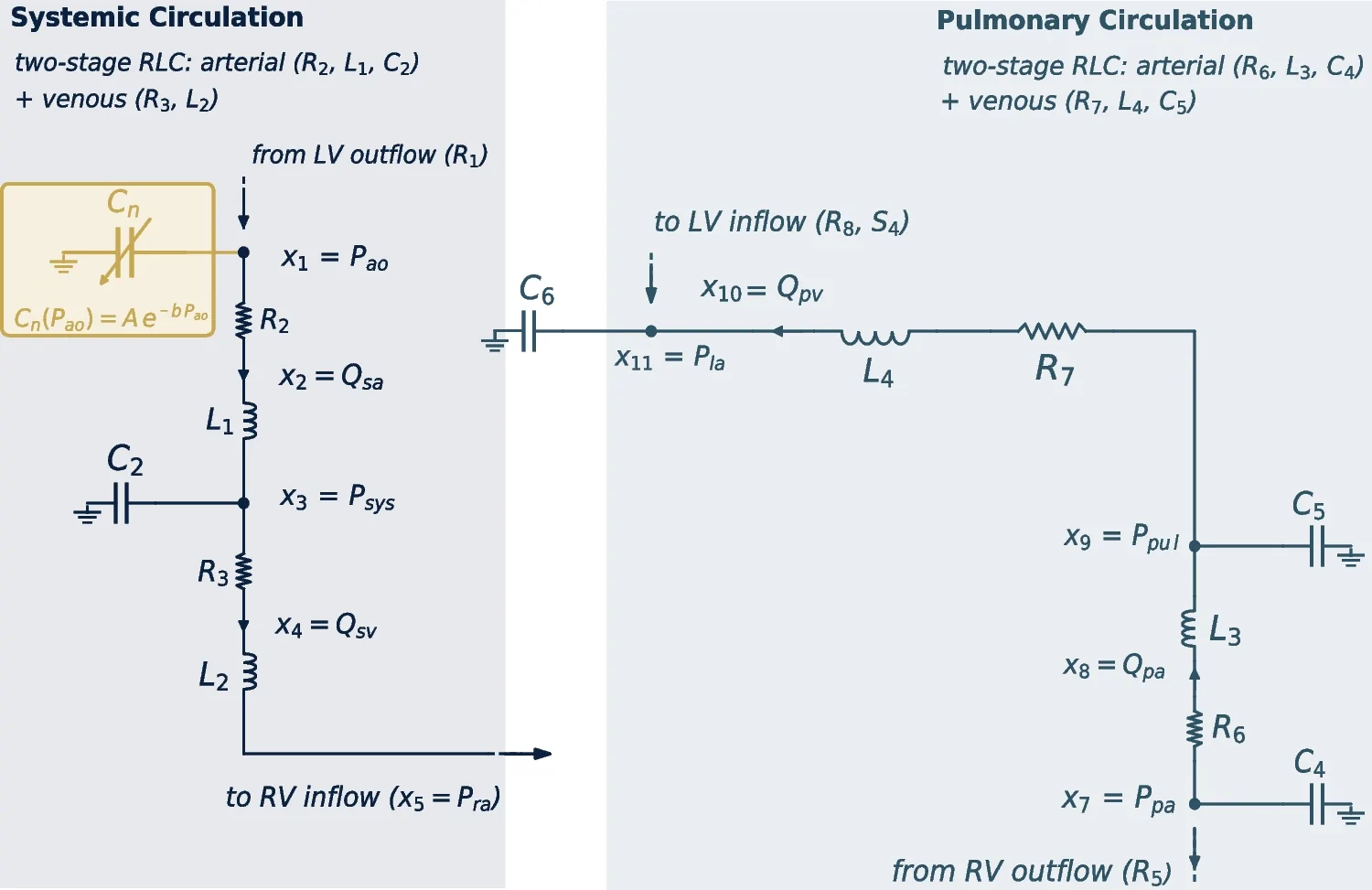
Electrical analogs of the systemic and pulmonary circulations, isolated from the closed-loop model in Fig. 2. Each circulation is represented as a two-stage RLC network comprising an arterial segment, an intermediate compliant node, and a venous segment. The systemic pathway links the aortic node $x_{1}=P_{\textrm{ao}}$ to the right atrial node $x_{5}=P_{\textrm{ra}}$ through $x_{3}=P_{\textrm{sys}}$, whereas the pulmonary pathway links $x_{7}=P_{\textrm{pa}}$ to the left atrial node $x_{11}=P_{\textrm{la}}$ through $x_{9}=P_{\textrm{pul}}$. The pressure-dependent aortic compliance $C_{n}(P_{\textrm{ao}})=A\,e^{-bP_{\textrm{ao}}}$ is the only nonlinear storage element and is highlighted in gold. All other capacitive elements shown here are linear. Dashed boundary segments indicate coupling to the ventricular subsystems

Systemic and pulmonary networks. The systemic and pulmonary circulations are represented by two-stage RLC subnetworks (Fig. 4). In each pathway, resistive elements account for viscous losses, inductive elements represent blood inertance, and capacitive elements describe compliant storage in the vessel walls. The high-pressure arterial portions are described by $C_n$ and $C_2$ in the systemic circulation and by $C_4$ and $C_5$ in the pulmonary circulation, whereas $C_3$ and $C_6$ represent the systemic and pulmonary venous reservoirs, respectively. Coupling between arterial and venous compartments occurs through the peripheral resistances $R_3$ and $R_7$, which set the main pressure losses and arterial-to-venous pressure gradients in the systemic and pulmonary beds.

#### State variables

The closed-loop dynamics is written as a system of twelve coupled first-order ODEs in state-space form. Let

$$ \textbf{x}(t)=\bigl [x_1(t),x_2(t),\dots ,x_{12}(t)\bigr ]^{\textsf{T}} $$

denote the state vector, whose components are pressures, flow rates, and ventricular volumes (Table 1). The model is written compactly as

8 $$\begin{aligned} \frac{d\textbf{x}}{dt} = \boldsymbol{\Phi }(\textbf{x},t), \end{aligned}$$

where $\boldsymbol{\Phi }=[\Phi _1,\Phi _2,\dots ,\Phi _{12}]^{\textsf{T}}$ collects the piecewise nonlinear right-hand sides obtained from KCL and KVL applied to the RLC network. This state-space form provides the basis for numerical integration, parameter sweeps, sensitivity analyses, and scenario-based simulations.

An explicit form of $\boldsymbol{\Phi }$ is given by

9 $$\begin{aligned} \begin{aligned} \frac{dx_1}{dt}&= \frac{1}{C_n}\left( \frac{S_1(t)\, \Delta P_1(t)}{R_L + R_1} - x_2 \right) , \\ \frac{dx_2}{dt}&= \frac{1}{L_1}\bigl (x_1 - R_2 x_2 - x_3\bigr ), \\ \frac{dx_3}{dt}&= \frac{1}{C_2}\bigl (x_2 - x_4\bigr ), \\ \frac{dx_4}{dt}&= \frac{1}{L_2}\bigl (x_3 - R_3 x_4 - x_5\bigr ), \\ \frac{dx_5}{dt}&= \frac{1}{C_3}\left( x_4 - \frac{S_2(t)\, \Delta P_2(t)}{R_4} \right) , \\ \frac{dx_6}{dt}&= \frac{S_2(t)\, \Delta P_2(t)}{R_4} - \frac{S_3(t)\, \Delta P_3(t)}{R_R + R_5}, \\ \frac{dx_7}{dt}&= \frac{1}{C_4}\left( \frac{S_3(t)\, \Delta P_3(t)}{R_R + R_5} - x_8 \right) , \\ \frac{dx_8}{dt}&= \frac{1}{L_3}\bigl (x_7 - R_6 x_8 - x_9\bigr ), \\ \frac{dx_9}{dt}&= \frac{1}{C_5}\bigl (x_8 - x_{10}\bigr ), \\ \frac{dx_{10}}{dt}&= \frac{1}{L_4}\bigl (x_9 - R_7 x_{10} - x_{11}\bigr ), \\ \frac{dx_{11}}{dt}&= \frac{1}{C_6}\left( x_{10} - \frac{S_4(t)\, \Delta P_4(t)}{R_8} \right) , \\ \frac{dx_{12}}{dt}&= \frac{S_4(t)\, \Delta P_4(t)}{R_8} - \frac{S_1(t)\, \Delta P_1(t)}{R_1 + R_L}. \end{aligned} \end{aligned}$$

The initial condition $\textbf{x}(0)$ is assigned from physiologically plausible estimates and is reported explicitly to ensure reproducibility.

In the linear baseline, $C_n$ is constant. In the nonlinear formulation, $C_n$ becomes pressure-dependent through an exponential law in $x_1$, consistent with previous nonlinear compliance descriptions in studies of systemic hemodynamics and hypertension [20–22, 29, 30]:

10 $$\begin{aligned} C_n(x_1)=A\,e^{-b x_1}. \end{aligned}$$

In Eq. (10), *A* is the aortic compliance scaling coefficient and *b* is the exponential stiffness coefficient. As $x_1$ increases, the effective compliance decreases, introducing a direct mechanism for pressure-dependent arterial stiffening within the closed-loop model. This construction preserves the low-order and physiologically interpretable structure of Windkessel-type descriptions.

### Numerical solution of the model equations

**Fig. 5:**
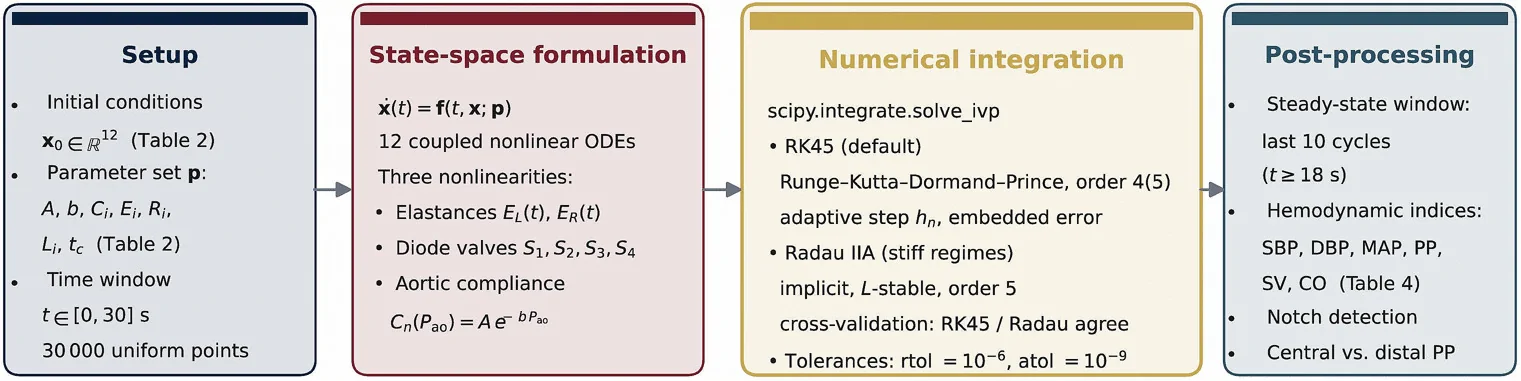
Simulation pipeline used for the numerical experiments reported in this work. The setup stage defines the initial state $\textbf{x}_{0}$ and parameter vector $\textbf{p}$, which feed the initial-value formulation in Eq. (11). The right-hand side includes the time-varying elastances, ideal-diode valve switching, and pressure-dependent aortic compliance described in Section 2.2. The gold-highlighted integration stage solves the ODE system with scipy.integrate.solve_ivp, using RK45 as the default solver and Radau IIA for stiffness-sensitive cross-validation. The post-processing stage computes the hemodynamic indices, detects the dicrotic notch, and evaluates the central-to-distal pulse-pressure ratio

The closed-loop cardiovascular model formulated in Section 2 yields a system of twelve coupled nonlinear ordinary differential equations (ODEs), written for numerical integration as

11 $$\begin{aligned} \frac{d\textbf{x}(t)}{dt} = \textbf{f}\!\left( t,\textbf{x}(t);\textbf{p}\right) , \qquad \textbf{x}(0) = \textbf{x}_0, \end{aligned}$$

where $\textbf{x}(t) \in \mathbb {R}^{12}$ is the state vector defined in Table 1, $\textbf{x}_0$ is the initial condition, and $\textbf{p}$ collects the fixed model parameters reported in Table 2. The right-hand side $\textbf{f}$ encodes the Kirchhoff balance equations of the lumped-parameter network. Three mechanisms introduce nonlinearity or non-smoothness into $\textbf{f}$: the time-varying ventricular elastances $E_L(t)$ and $E_R(t)$, the switching of the ideal-diode valves $S_1$ through $S_4$, and the pressure-dependent aortic compliance $C_n(P_{\textrm{ao}}) = A\,e^{-bP_{\textrm{ao}}}$ defined in Eq. (10). The trajectory $\textbf{x}(t)$ is then computed numerically. Figure 5 summarizes the complete simulation pipeline, from initialization to post-processing.

**Table 2:** Initial conditions and fixed parameters of the closed-loop cardiovascular model. State variables $x_{i}$ are annotated with their physical symbols (*P*, *Q*, or *V*) for consistency with Figs. 1 through 4. Pressures are given in mmHg, and all remaining quantities follow the mmHg-based unit convention

Parameter	Description	Value
Initial conditions of the twelve-state variables		
$x_{1_{(0)}}\;(P_{\textrm{ao}})$	Initial aortic pressure [mmHg]	71.112
$x_{2_{(0)}}\;(Q_{\textrm{sa}})$	Initial systemic arterial flow [cm$^3$ s$^{-1}$]	8.880
$x_{3_{(0)}}\;(P_{\textrm{sys}})$	Initial systemic pressure [mmHg]	70.52
$x_{4_{(0)}}\;(Q_{\textrm{sv}})$	Initial systemic venous flow [cm$^3$ s$^{-1}$]	67.34
$x_{5_{(0)}}\;(P_{\textrm{ra}})$	Initial right atrial pressure [mmHg]	3.33
$x_{6_{(0)}}\;(V_{RV})$	Initial right ventricular volume [cm$^3$]	105.52
$x_{7_{(0)}}\;(P_{\textrm{pa}})$	Initial pulmonary arterial pressure [mmHg]	13.42
$x_{8_{(0)}}\;(Q_{\textrm{pa}})$	Initial pulmonary arterial flow [cm$^3$ s$^{-1}$]	0.786
$x_{9_{(0)}}\;(P_{\textrm{pul}})$	Initial pulmonary capillary pressure [mmHg]	13.40
$x_{10_{(0)}}\;(Q_{\textrm{pv}})$	Initial pulmonary venous flow [cm$^3$ s$^{-1}$]	23.84
$x_{11_{(0)}}\;(P_{\textrm{la}})$	Initial left atrial pressure [mmHg]	11.30
$x_{12_{(0)}}\;(V_{LV})$	Initial left ventricular volume [cm$^3$]	112.76
Nonlinear aortic compliance parameters $C_n(P_{\textrm{ao}}) = A\, e^{-b\, P_{\textrm{ao}}}$		
*A*	Aortic compliance scaling factor [cm$^3$ mmHg$^{-1}$]	1.470
*b*	Aortic stiffness coefficient [mmHg$^{-1}$]	0.023
Linear compliances of the systemic and pulmonary circulations		
$C_2$	Systemic arterial compliance [cm$^3$ mmHg$^{-1}$]	1.460
$C_3$	Systemic venous compliance [cm$^3$ mmHg$^{-1}$]	20.00
$C_4$	Pulmonary arterial compliance [cm$^3$ mmHg$^{-1}$]	$9.000 \times 10^{-2}$
$C_5$	Pulmonary capillary compliance [cm$^3$ mmHg$^{-1}$]	2.670
$C_6$	Pulmonary venous compliance [cm$^3$ mmHg$^{-1}$]	46.70
Ventricular time-varying elastances		
$E_{dL}$	Left ventricular elastance, diastole [mmHg cm$^{-3}$]	0.100
$E_{sL}$	Left ventricular elastance, systole [mmHg cm$^{-3}$]	1.375
$E_{dR}$	Right ventricular elastance, diastole [mmHg cm$^{-3}$]	0.030
$E_{sR}$	Right ventricular elastance, systole [mmHg cm$^{-3}$]	0.328
Inertances of the systemic and pulmonary segments		
$L_1$	Aortic-systemic inertance [mmHg s$^2$ cm$^{-3}$]	$8.251 \times 10^{-4}$
$L_2$	Systemic venous inertance [mmHg s$^2$ cm$^{-3}$]	$3.600 \times 10^{-3}$
$L_3$	Pulmonary arterial inertance [mmHg s$^2$ cm$^{-3}$]	$7.501 \times 10^{-4}$
$L_4$	Pulmonary venous inertance [mmHg s$^2$ cm$^{-3}$]	$3.080 \times 10^{-3}$
Resistances: valvular, vascular, and myocardial		
$R_L$	Left myocardial viscosity [mmHg s cm$^{-3}$]	0.0800
$R_R$	Right myocardial viscosity [mmHg s cm$^{-3}$]	0.0175
$R_1$	Aortic valve resistance [mmHg s cm$^{-3}$]	$3.751 \times 10^{-3}$
$R_2$	Aortic-systemic resistance [mmHg s cm$^{-3}$]	0.0675
$R_3$	Systemic peripheral resistance [mmHg s cm$^{-3}$]	1.000
$R_4$	Tricuspid valve resistance [mmHg s cm$^{-3}$]	$3.751 \times 10^{-3}$
$R_5$	Pulmonary valve resistance [mmHg s cm$^{-3}$]	$3.751 \times 10^{-3}$
$R_6$	Pulmonary arterial resistance [mmHg s cm$^{-3}$]	$3.761 \times 10^{-3}$
$R_7$	Pulmonary peripheral resistance [mmHg s cm$^{-3}$]	0.1013
$R_8$	Mitral valve resistance [mmHg s cm$^{-3}$]	$3.751 \times 10^{-3}$
Other parameters		
$\textrm{Ref}$	Aortic valve reflux [%]	0
$t_c$	Cardiac period [s]	0.800

An adaptive explicit Runge-Kutta method of order *p* approximates the solution between successive instants $t_n$ and $t_{n+1}=t_n+h_n$ by combining intermediate evaluations of $\textbf{f}$ within each step. The corresponding local truncation error scales as $O(h_n^{p+1})$ [31]. The error is estimated from an embedded lower-order approximation computed from the same function evaluations, and the step size $h_n$ is adjusted to keep the estimate below the prescribed tolerance. The integrator uses larger steps in smooth segments of the trajectory and refines automatically near valve transitions, where $\textbf{f}$ changes abruptly.

Two complementary solvers are used. The default **RK45** method is the explicit Runge-Kutta-Dormand-Prince scheme of order 4(5) [32, 33], implemented through scipy.integrate.solve_ivp [34]. This method is well suited to the smooth portions of the dynamics away from valve transitions. For the stiffness-dominated behavior observed at the largest values of *b* in the sensitivity sweep (Section 3.2), the simulations are cross-checked with the implicit, *L*-stable **Radau IIA** method of order 5 [35], which is appropriate for systems with separated timescales. The two solvers produce indistinguishable hemodynamic indices within the reported precision in all scenarios considered, supporting the interpretation that the results reflect the model dynamics rather than artifacts of the integration scheme. Tolerances are set to $\textrm{rtol} = 10^{-6}$ and $\textrm{atol} = 10^{-9}$ throughout, which is conservative for the magnitudes of the state variables (Table 2).

The trajectory is sampled at *30 000* uniformly spaced points over a 30-second window, corresponding to approximately 37.5 cardiac cycles at the nominal period $t_c = 0.8$ s and about 800 samples per cycle. Steady-state averages of SBP, DBP, MAP, PP, SV, and CO are computed over the last ten cycles to reduce the influence of the initial transient. The dicrotic notch is detected automatically in each steady-state cycle by locating the first local minimum of $P_{\textrm{ao}}(t)$ between the systolic peak and the subsequent diastolic secondary peak. This detection uses the find_peaks routine of SciPy [34], with minimum-distance and prominence constraints set according to the cardiac timescale. The central-to-distal pulse-pressure ratio in Section 3.2 is computed cycle by cycle from $P_{\textrm{ao}}(t)$ and $P_{\textrm{sys}}(t)$ and reported as the mean over the steady-state window.

The complete pipeline is implemented in Python 3 using NumPy [36], SciPy [34], and Matplotlib [37], following established practices for scientific computing and dynamical-systems simulation in Python [38, 39]. The ODE system is integrated through scipy.integrate.solve_ivp [34].

### Baseline parameter set

The baseline values of the model parameters used throughout this work are collected in Table 2 and are organized by category. The systemic and pulmonary RLC parameters along with the ventricular elastance profiles are based on established closed-loop cardiovascular-modeling formulations, following Avanzolini et al. [16] and Halaimi et al. [23]. The adopted diastolic and systolic elastance levels are also consistent with those reported by Diourté et al. [30]. Together, these choices define a resting adult operating point with hemodynamic indices within commonly reported physiological ranges.

The parameters *A* and *b* define the nonlinear aortic compliance law $C_n(P_{\textrm{ao}}) = A\,e^{-bP_{\textrm{ao}}}$ in Eq. (10). They are calibrated to match the linear three-element Windkessel parameterization of Cappello et al. [21] at the nominal aortic pressure $P_{0} = 80$ mmHg, such that $C_n(P_{0})$ equals the linear compliance used as the comparison baseline in Table 3. This calibration yields *A = 1.470* cm$^{3}$ mmHg$^{-1}$ and *b = 0.023* mmHg$^{-1}$. Consequently, the linear and nonlinear formulations share the same instantaneous compliance at the nominal operating point and diverge progressively as $P_{\textrm{ao}}$ departs from $P_{0}$. This construction isolates the effect of pressure dependence from differences in nominal compliance.

**Table 3:** Hemodynamic indices computed over the last ten steady-state cardiac cycles for the linear baseline, three nonlinear aortic-stiffness cases, and two pathological scenarios. Columns report systolic (SBP) and diastolic (DBP) blood pressure, mean arterial pressure (MAP), pulse pressure (PP), stroke volume (SV), and cardiac output (CO). Pressure indices are extracted from $x_{1}=P_{\textrm{ao}}$ and stroke volume from $x_{12}=V_{\textrm{LV}}$. Pressures are reported in mmHg, SV in cm$^{3}$, and CO in L min$^{-1}$

Scenario	SBP	DBP	MAP	PP	SV / CO
Sensitivity to the aortic-stiffness coefficient *b*					
Linear baseline ($$C_n = $$ const.)	112.93	68.48	86.22	44.45	61.91 / 4.64
Nonlinear nominal ($C_n(P_{\textrm{ao}})$, *b=0.023*)	113.79	68.63	86.24	45.16	61.92 / 4.64
Compliant aorta (*b = 0.010* mmHg$^{-1}$)	108.96	72.69	86.87	36.27	62.38 / 4.68
Stiffened aorta (*b = 0.040* mmHg$^{-1}$)	115.71	66.47	86.04	49.24	61.77 / 4.63
Pathological scenarios					
Aortic regurgitation (5% reflux)	111.73	57.91	78.85	53.82	75.40 / 5.66
Arteriosclerosis (*-32%* *A*, *+75%* *b*)	115.90	66.15	86.01	49.75	61.75 / 4.63

The initial condition $\textbf{x}_{0}$ listed in Table 2 defines a physiologically plausible resting operating point. The results reported below are computed as steady-state averages over the last ten cardiac cycles (Section 2.2), after discarding the initial transient. Perturbations of $\textbf{x}_{0}$ by up to $\pm 10\%$ produced the same steady-state indices in Table 3 within the resolution of the post-processing routines. This robustness indicates that the reported operating point lies on a stable attracting regime for the parameter set considered.

## Results

### Baseline dynamics and hemodynamic indices

**Fig. 6:**
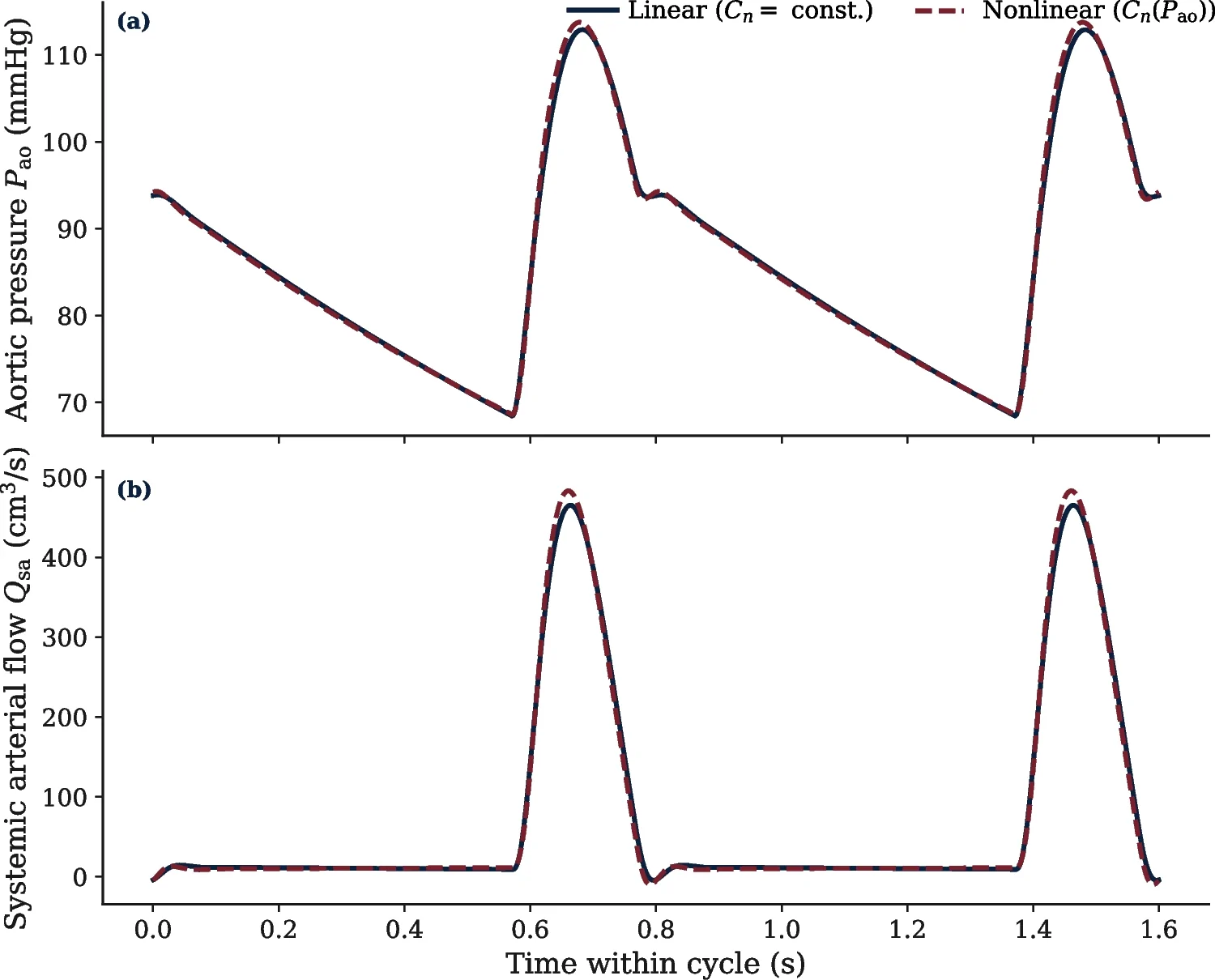
Aortic pressure $x_{1}$ and systemic arterial flow $x_{2}$ during the last two steady-state cardiac cycles. Linear and nonlinear responses are shown under identical initial conditions and numerical settings (RK45, $\textrm{rtol} = 10^{-6}$, $\textrm{atol} = 10^{-9}$)

**Fig. 7:**
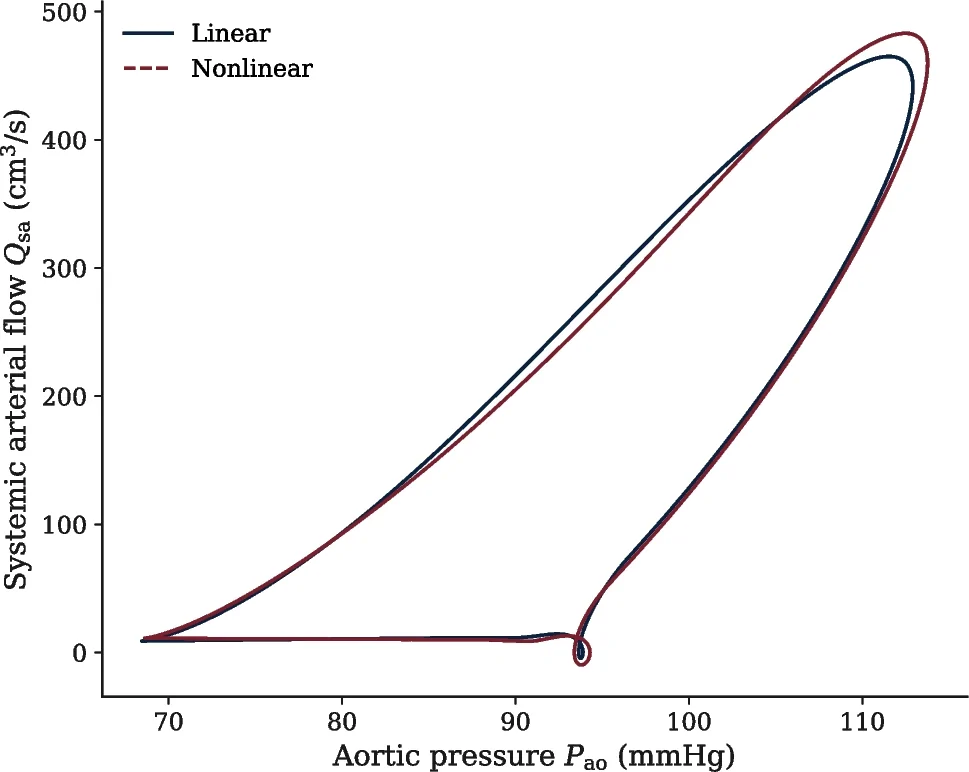
Phase portrait in the $(x_{1},x_{2})$ plane for the linear and nonlinear responses under nominal conditions, computed over the last five cardiac cycles. The nonlinear trajectory is slightly broader, particularly along the systolic ejection branch

Under nominal conditions, the pressure-dependent compliance law preserves the linear baseline at both waveform and phase-space levels (Figs. 6 and 7). Aortic pressure $x_{1}$ and systemic arterial flow $x_{2}$ remain nearly superimposable between the two formulations, with only small differences in their extrema. The corresponding trajectory in the $(x_{1},x_{2})$ plane is slightly broader in the nonlinear case, mainly along the systolic ejection branch. This agreement provides a baseline consistency check: the nonlinear formulation recovers the established linear response near the nominal operating point while retaining the ability to express larger departures when the compliance law is probed away from that regime.

To complement the waveform-level comparison, standard hemodynamic indices were computed from the last ten cardiac cycles of each simulation, using the proximal aortic pressure $x_{1}$ as the reference signal (Table 3). The adaptive explicit solver and the implicit Radau IIA scheme produce indistinguishable indices, within the reported precision, at both the aortic node $x_{1}$ and the systemic node $x_{3}$. This agreement indicates that the reported values are robust with respect to step-size control, stiffness handling, and valve-switching events.

From a hemodynamic perspective, the pressure-dependent aortic compliance law $C_n(x_{1}) = A\,e^{-b x_{1}}$ produces a coherent stiffness-mediated modulation of proximal pulsatility while mean loading and global pump output remain comparatively preserved. At the nominal operating point, the linear and nonlinear formulations differ by approximately 1 mmHg or less in the pressure indices reported in Table 3. Across the *b* sweep, the compliant and stiffer regimes shift proximal pulse pressure by approximately $\Delta \textrm{PP}(x_{1}) \approx 12.97$ mmHg, while SV remains in the range 61.77 to 62.38 cm$^{3}$ and CO remains in the range 4.63 to 4.68 L/min. This pattern is consistent with classical arterial mechanics: compliance primarily modulates pulsatility, whereas mean pressure and pump output are less sensitive when peripheral resistance and ventricular driving are held fixed [27, 28, 40].

### Stiffness sensitivity and ventricular pressure-volume loops

Varying the exponential stiffness coefficient *b* in Eq. (10) reveals how pressure-dependent aortic compliance modulates the hemodynamic response and separates proximal from distal pressure behavior within the lumped formulation. This comparison is motivated by classical studies linking arterial compliance, pulse pressure, and central-to-peripheral pressure differences [28, 40].

**Fig. 8:**
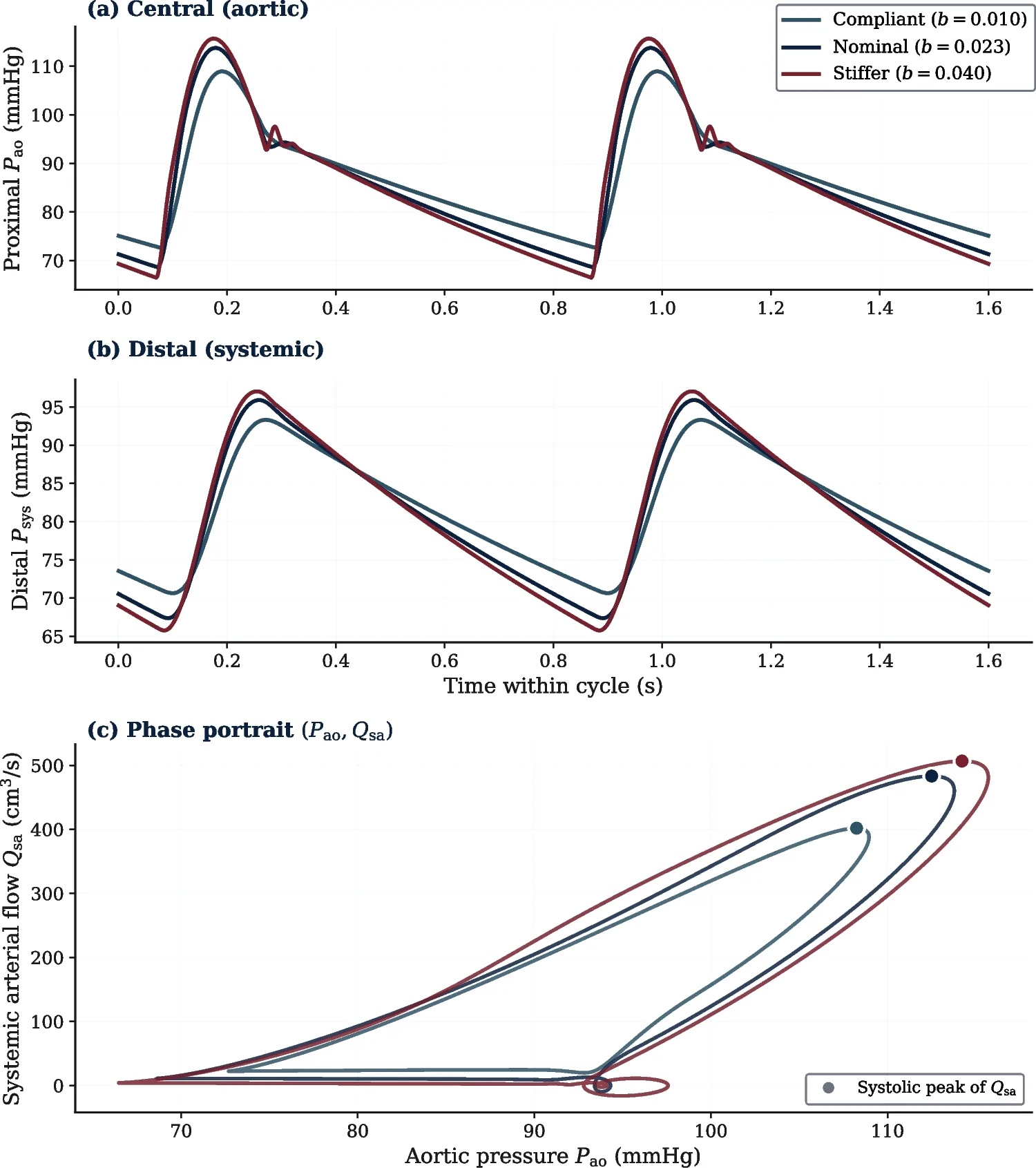
Stiffness sensitivity of the cardiovascular response to the aortic-stiffness coefficient *b* in $C_{n}(P_{\textrm{ao}})=A\,e^{-bP_{\textrm{ao}}}$. Three regimes are compared: compliant (*b=0.010* mmHg$^{-1}$), nominal (*b=0.023* mmHg$^{-1}$), and stiffer (*b=0.040* mmHg$^{-1}$). Panels (a) and (b) show proximal aortic pressure $x_{1}=P_{\textrm{ao}}$ and distal systemic pressure $x_{3}=P_{\textrm{sys}}$ over two steady-state cardiac cycles. Panel (c) shows the corresponding $(x_{1},x_{2})$ phase portrait over one cycle, with the systolic $Q_{\textrm{sa}}$ peak marked for each regime. Increasing *b* widens proximal pulsatility and broadens the limit cycle; the distal response remains attenuated, and small diastolic structures reflect altered pressure-flow timing within the 0D network

Figure 8 shows the resulting changes in proximal aortic pressure $x_{1}$, distal systemic pressure $x_{3}$, and the $(x_{1},x_{2})$ phase portrait. The compliant regime (*b = 0.010* mmHg$^{-1}$) reduces proximal pulsatility, with lower SBP, higher DBP, and smaller temporal excursions. The stiffer regime (*b = 0.040* mmHg$^{-1}$) increases pulse pressure, sharpens the systolic and diastolic transitions, and broadens the phase-space trajectory. Small secondary structures near diastole indicate altered pressure-flow timing within the lumped network, rather than spatially resolved wave reflection. The systolic peak markers in the phase portrait shift along a stiffness-ordered direction, showing that the nonlinear compliance reshapes the limit cycle selectively instead of producing a uniform amplitude increase. These trends are consistent with classical observations that arterial compliance strongly affects pulse pressure and wave-related hemodynamic indices [27, 41].

**Fig. 9:**
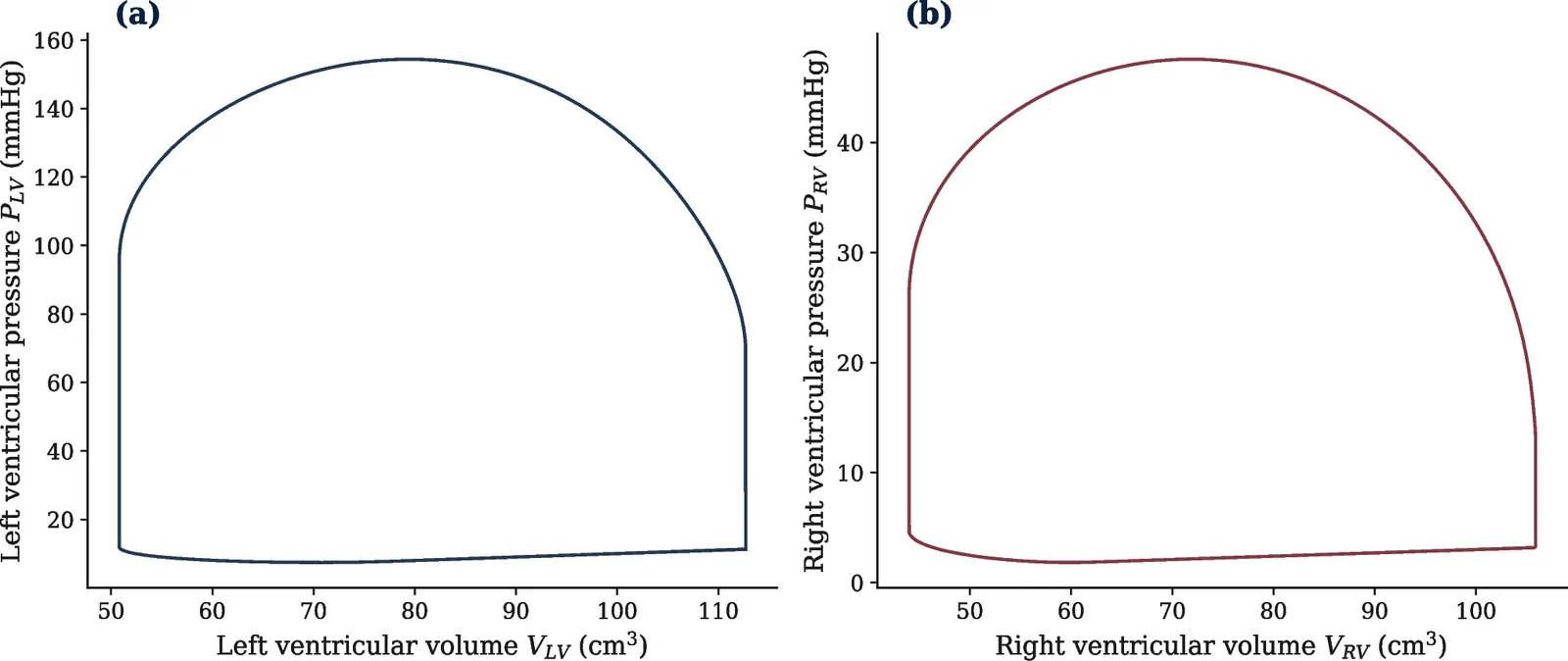
Steady-state pressure-volume loops reconstructed from the nominal nonlinear simulation. The left-ventricular loop spans 50 to 113 cm$^{3}$ and reaches $P_{\textrm{LV},\max }\approx 165$ mmHg, whereas the right-ventricular loop spans 52 to 105 cm$^{3}$ and reaches $P_{\textrm{RV},\max }\approx 40$ mmHg. The last five cardiac cycles show no detectable cycle-to-cycle drift

The stiffness analysis is complemented by the ventricular pressure-volume loops reconstructed under the nominal nonlinear calibration (Fig. 9). These loops provide an independent cardiac-level check of the closed-loop response, because their geometry emerges from the interaction among ventricular elastance, valve switching, and upstream and downstream vascular compartments.

The reconstructed left-ventricular loop exhibits the four canonical phases of the cardiac cycle: isovolumetric contraction, ejection, isovolumetric relaxation, and filling. It yields $\textrm{SV}\approx 63$ cm$^{3}$ and a peak pressure near 165 mmHg. The right-ventricular loop shows the analogous four-phase morphology, with a comparable stroke volume in the expected lower-pressure range ($P_{\textrm{RV},\max }\approx 40$ mmHg). Both loops are consistent with ranges reported by reference closed-loop lumped-parameter models [14, 16] and with resting adult pressure-volume behavior. They also reproduce qualitative features commonly used in the interpretation of clinical *in vivo* PV-loops, including a distinct end-systolic region, a low-pressure filling branch, and a clearly separated isovolumetric contraction segment. This consistency is a non-trivial outcome of the closed-loop coupling: the loop geometry emerges from the interaction among time-varying elastance, valve switching, and the upstream and downstream lumped compartments, rather than being prescribed directly.

### Dicrotic notch in the aortic pressure waveform

**Fig. 10:**
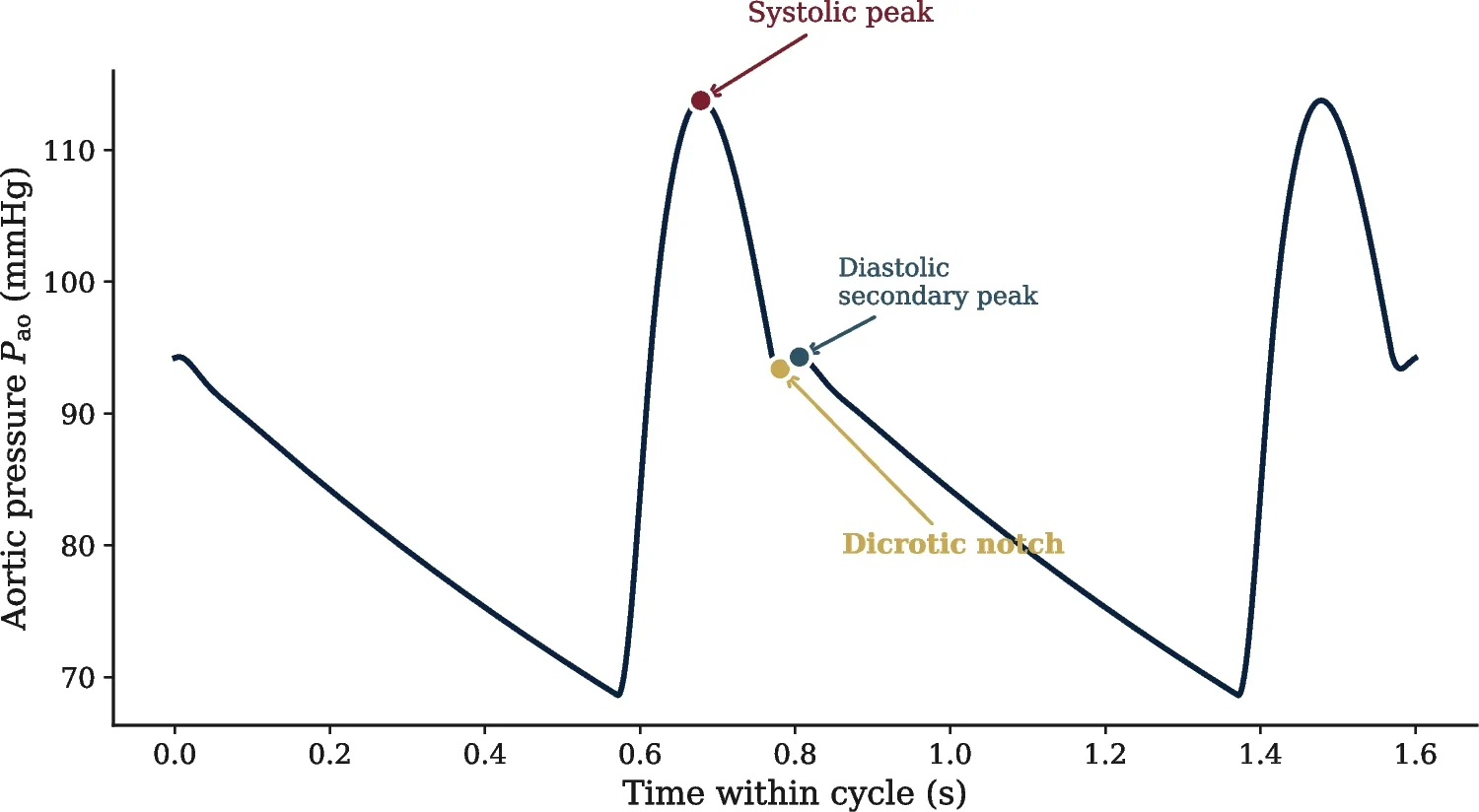
Dicrotic notch reproduced in the steady-state aortic pressure waveform of the nonlinear lumped model. The systolic peak, notch, and diastolic secondary peak are annotated in the second displayed cardiac cycle to highlight the post-ejection waveform structure. The notch occurs approximately *Δ t=0.07* s after the systolic peak and reaches about 95 mmHg

Under nominal conditions, the aortic pressure waveform exhibits a clear dicrotic notch followed by a diastolic secondary rise (Fig. 10). The notch is identified algorithmically as the first local minimum after the systolic peak (Section 2.2). In the steady-state waveform, it occurs approximately 0.07 s after the systolic peak, with a timing consistent with the reported post-ejection interval of 0.05 to 0.15 s for the dicrotic notch [41].

Mechanistically, the notch is associated with aortic valve closure. When ventricular ejection terminates, the abrupt change in outflow produces a localized decrease in proximal aortic pressure, followed by partial recovery through elastic recoil and inertial redistribution within the lumped network. The reproduction of this feature in a single-node 0D representation is consistent with prior observations that valve switching combined with lumped inertance can reproduce both the notch and the secondary pressure rise without explicit 1D wave propagation [27]. The notch should therefore not be interpreted, by itself, as evidence of spatially resolved wave dynamics. Instead, its persistence under the nonlinear compliance law indicates that the proposed pressure-dependent element preserves a temporal waveform feature commonly used in clinical aortic-pressure assessment.

### Physiological challenges: afterload, heart rate, contractility, and venous return

To probe the closed-loop response of the nonlinear model under physiological perturbations, four scenarios were simulated by changing one parameter at a time relative to the nominal nonlinear baseline. The first three follow the standard sensitivity protocol used in reference closed-loop studies [14, 16]: increased afterload, implemented by raising $R_3$ by *40%*; increased heart rate, implemented by reducing $t_c$ from 0.8 to 0.6 s, corresponding to 75 to 100 bpm; and increased left-ventricular contractility, implemented by raising $E_{s,L}$ from 1.375 to 1.8 mmHg cm$^{-3}$, approximately *30%* above baseline. The fourth scenario emulates reduced venous return by increasing the systemic venous compliance $C_3$ by *150%* relative to baseline, thereby sequestering a larger fraction of the total blood volume in the venous reservoir as a lumped-parameter analog of postural venous pooling [42].

**Fig. 11:**
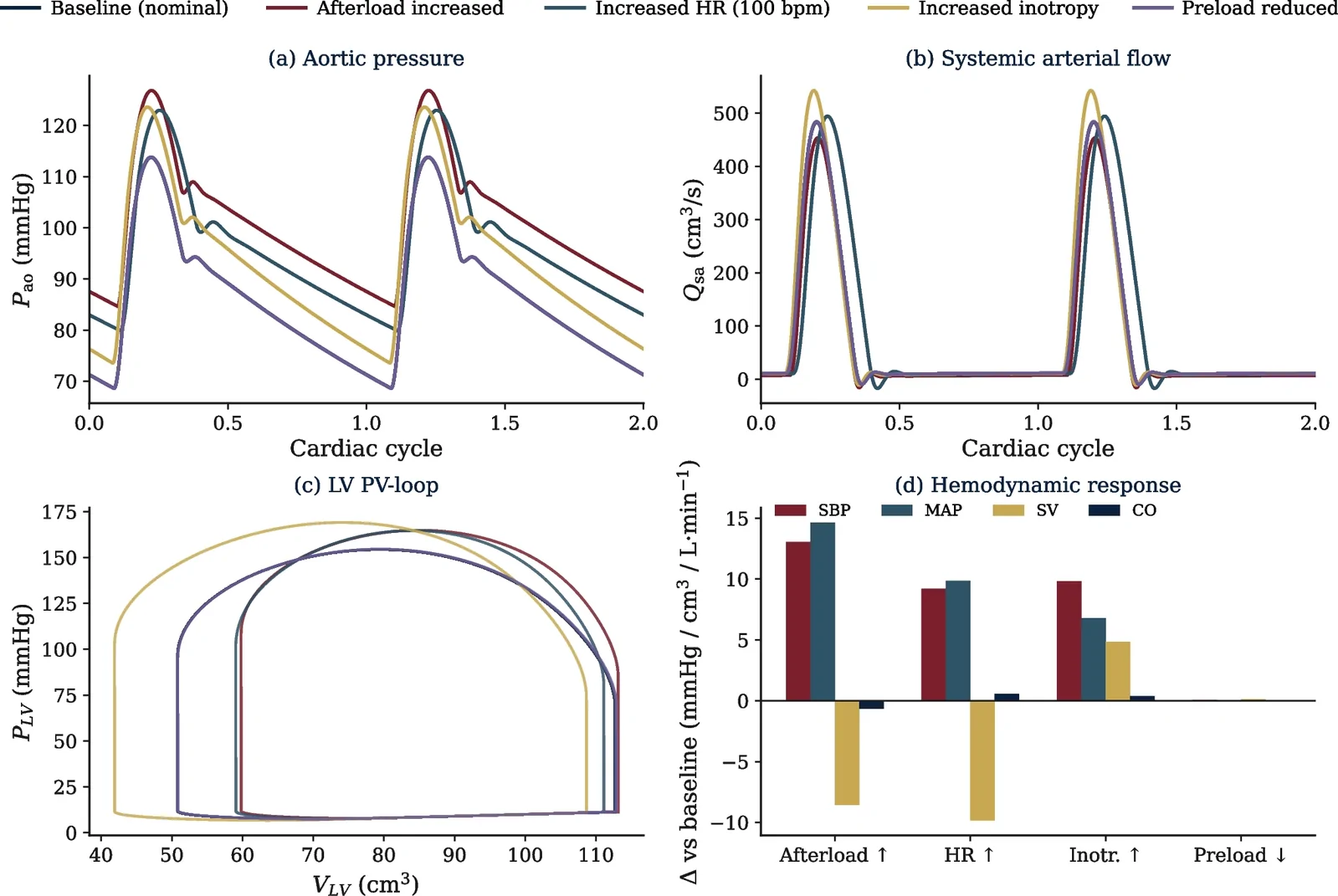
Hemodynamic response to four physiological challenges, evaluated relative to the nominal nonlinear baseline. Panels (a) and (b) compare aortic pressure and systemic arterial flow over two cardiac cycles using normalized cardiac-cycle phase, which aligns scenarios with different periods $t_c$. Panel (c) shows the associated steady-state left-ventricular pressure-volume loops, and panel (d) reports the baseline-relative changes in SBP, MAP, SV, and CO

Figure 11 compares the four perturbations with the nominal baseline through four observables: aortic pressure, systemic arterial flow, the left-ventricular pressure-volume loop, and baseline-relative changes in SBP, MAP, SV, and CO. Panels (a) and (b) use normalized cardiac cycle phase to align scenarios with different cardiac periods $t_c$, so that the same horizontal interval corresponds to one full cardiac cycle in each case.

The qualitative signature of each perturbation follows the expected direction of response. The afterload increase produces a hypertensive shift, with SBP and MAP elevated by approximately 13 mmHg and a moderate reduction in SV, consistent with the higher impedance encountered during ejection. CO remains close to baseline, suggesting a limited Frank-Starling-type adjustment within the closed-loop coupling rather than an explicitly regulated compensatory response. The heart-rate challenge raises SBP and MAP by approximately 9 and 10 mmHg, respectively, while SV decreases by about 10 cm$^{3}$ due to the shorter filling time. On a per-minute basis, CO remains close to baseline because the reduction in SV is offset by the higher beat frequency. The contractility challenge raises SBP and increases SV by approximately 5 cm$^{3}$, producing a wider and taller left-ventricular PV-loop in panel (c).

The venous-return reduction challenge produces the weakest global response, with SBP, MAP, SV, and CO remaining close to their baseline values. This limited shift is consistent with the closed-loop structure used here, which does not include explicit autonomic regulation. Increasing $C_3$ redistributes part of the blood volume toward the systemic venous reservoir, but the resulting perturbation is largely accommodated by the attracting limit-cycle dynamics through ventricular filling, valve-mediated flow, and the compliant storage elements of the network. Consequently, the waveforms change locally without a sustained displacement of the mean operating point. A stronger orthostatic-like response, including larger reductions in SV and MAP, would require additional mechanisms such as explicit blood-volume redistribution, regulatory feedback, or compartmental removal from the systemic circulation, which are outside the scope of the present study.

**Fig. 12:**
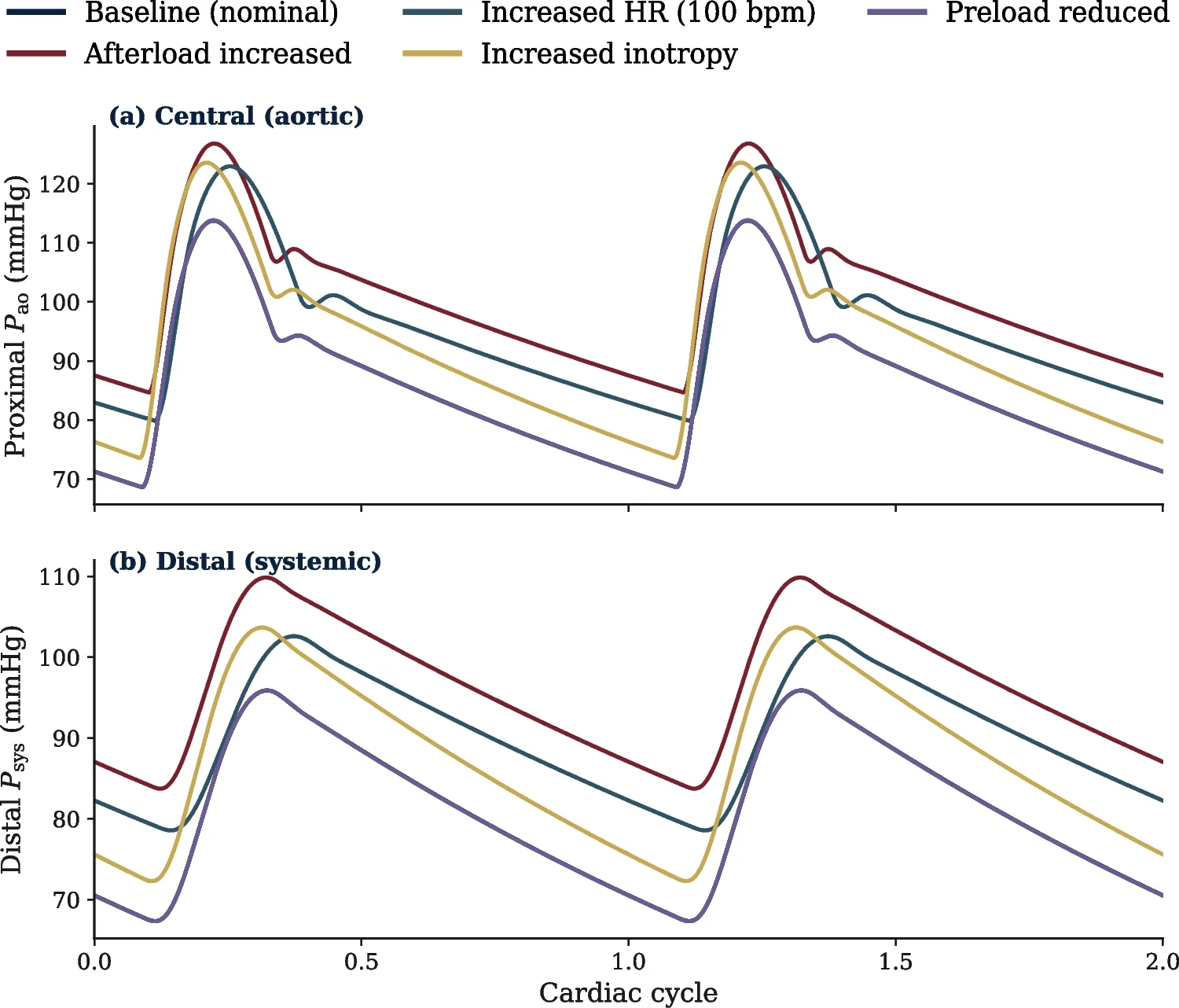
Central-to-distal pressure response across the four physiological challenges. Panels (**a**) and (**b**) compare proximal aortic pressure $x_{1}$ and distal systemic pressure $x_{3}$ over two cardiac cycles using normalized cardiac-cycle phase. Across all challenges, the larger proximal pressure excursions indicate that the pressure-dependent aortic compliance primarily modulates pulsatility at the central arterial node within the present 0D formulation

The proximal-to-distal distinction introduced in Section 3.2 also appears in the physiological challenge scenarios. Figure 12 compares the proximal aortic pressure $x_{1}$ and distal systemic pressure $x_{3}$ across the four perturbations. The larger pressure excursions at $x_{1}$ indicate that the pressure-dependent compliance acts primarily on central arterial pulsatility, while the downstream systemic elements attenuate this effect before it reaches the distal pressure node within the 0D network.

### Robustness checks: pulmonary nonlinearity and solver consistency

A natural follow-up question is whether the pressure-dependent compliance law should also be introduced in the pulmonary circulation. The form $C_n(P)=A\,e^{-bP}$ is not specific to the systemic bed; rather, its effective contribution depends on the pressure range over which the nonlinearity is exercised. To assess whether restricting the nonlinear element to the aortic branch affects the systemic conclusions, the same exponential law was applied to the pulmonary compliant compartment $C_5$ over $b_{\textrm{pul}}\in \{0.010,\,0.023,\,0.040\}$ mmHg$^{-1}$, mirroring the systemic sweep.

For each value of $b_{\textrm{pul}}$, the scaling coefficient $A_{\textrm{pul}}$ was calibrated so that $C_n(P_{\textrm{ref}})$ at $P_{\textrm{ref}}=15$ mmHg matched the nominal linear value $C_5=2.67$ cm$^{3}$ mmHg$^{-1}$. This calibration isolates the effect of the pressure-dependent shape of the compliance law from any change in its operating-point value. The test therefore evaluates the modeling choice itself, rather than a simple rescaling of pulmonary compliance.

**Fig. 13:**
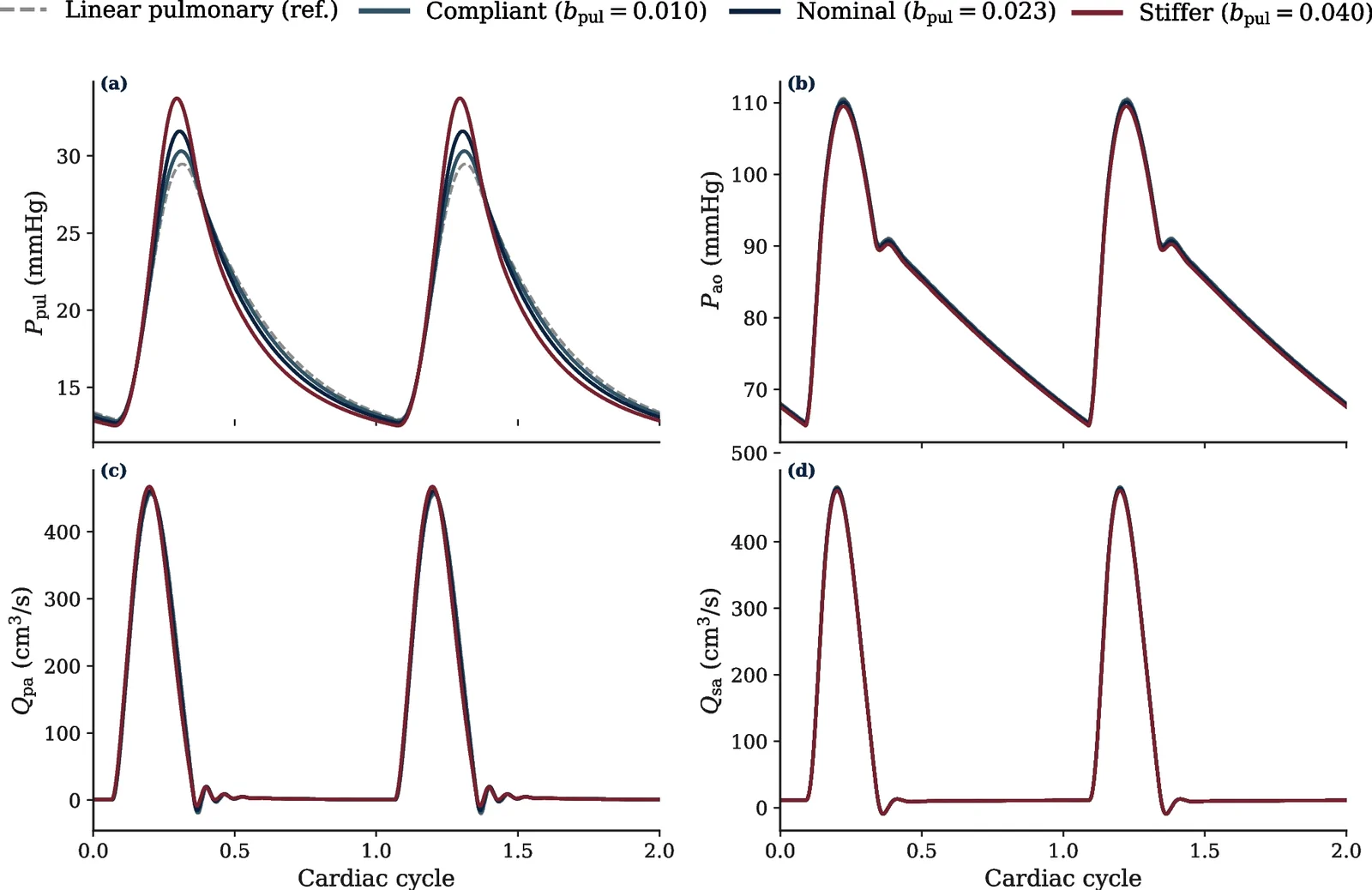
Robustness test for the pulmonary pressure-dependent compliance. The left column compares pulmonary capillary pressure $P_{\textrm{pul}}$ and pulmonary arterial flow $Q_{\textrm{pa}}$ across three values of the pulmonary-stiffness coefficient $b_{\textrm{pul}}$ and the linear pulmonary reference. The right column reports the corresponding systemic aortic pressure $P_{\textrm{ao}}$ and systemic arterial flow $Q_{\textrm{sa}}$. The near-superimposable systemic responses indicate negligible spillover from the pulmonary nonlinearity to systemic hemodynamics

Two observations follow from Fig. 13. First, the pulmonary pressure range across the cardiac cycle is narrow, approximately 15 to 28 mmHg. Consequently, the multiplicative factor $e^{-b_{\textrm{pul}}\Delta P}$ varies only modestly along the orbit, even for the largest value of $b_{\textrm{pul}}$ considered. The resulting pulmonary pulse-pressure modulation is on the order of 1 mmHg, more than an order of magnitude smaller than the approximately 13 mmHg modulation observed in the systemic bed over the analogous range of *b* (Table 3, sensitivity rows). Second, the systemic waveforms in panels (b) and (d) remain nearly superimposable across the pulmonary sweep, indicating negligible spillover from the pulmonary nonlinearity to systemic hemodynamics.

Taken together, these results support the modeling choice of applying the pressure-dependent compliance law only to the systemic circulation in the present study. Because the pulmonary bed operates over a much lower pressure range, extending the same nonlinear law to that side adds model complexity with little effect on the systemic responses analyzed here. Keeping the nonlinearity localized in the aortic branch also preserves a clear interpretation of the *b* sensitivity sweep.

A complementary robustness check concerns the numerical integration scheme. For the regimes explored here, the adaptive explicit Runge-Kutta solver and the implicit Radau IIA method converge to indistinguishable steady-state indices within the reported precision (Table 3). This agreement supports the interpretation that the reported hemodynamic values reflect the model dynamics rather than artifacts of step-size control, stiffness handling, or valve switching. The diagnostic relevance of this solver comparison for stiffness-aware sensitivity analysis is discussed in Section 4.

### Pathophysiological emulation via targeted parameter modifications

Beyond parameter sensitivity, the nonlinear framework was used to emulate two aorta-related pathophysiological signatures while preserving the same closed-loop architecture. Aortic regurgitation was represented by setting the aortic-valve reflux parameter to 0.05 and reducing the aortic-valve resistance $R_1$ by *60%*, whereas arterial stiffening was represented by reducing the aortic compliance scaling coefficient *A* by *32%* and increasing the exponential stiffness coefficient *b* by *75%* (Table 3, last two rows). These localized parameter changes are not intended to reproduce the full clinical complexity of each condition but to test whether the model generates qualitatively consistent waveform and index-level responses.

#### Aortic regurgitation

**Fig. 14:**
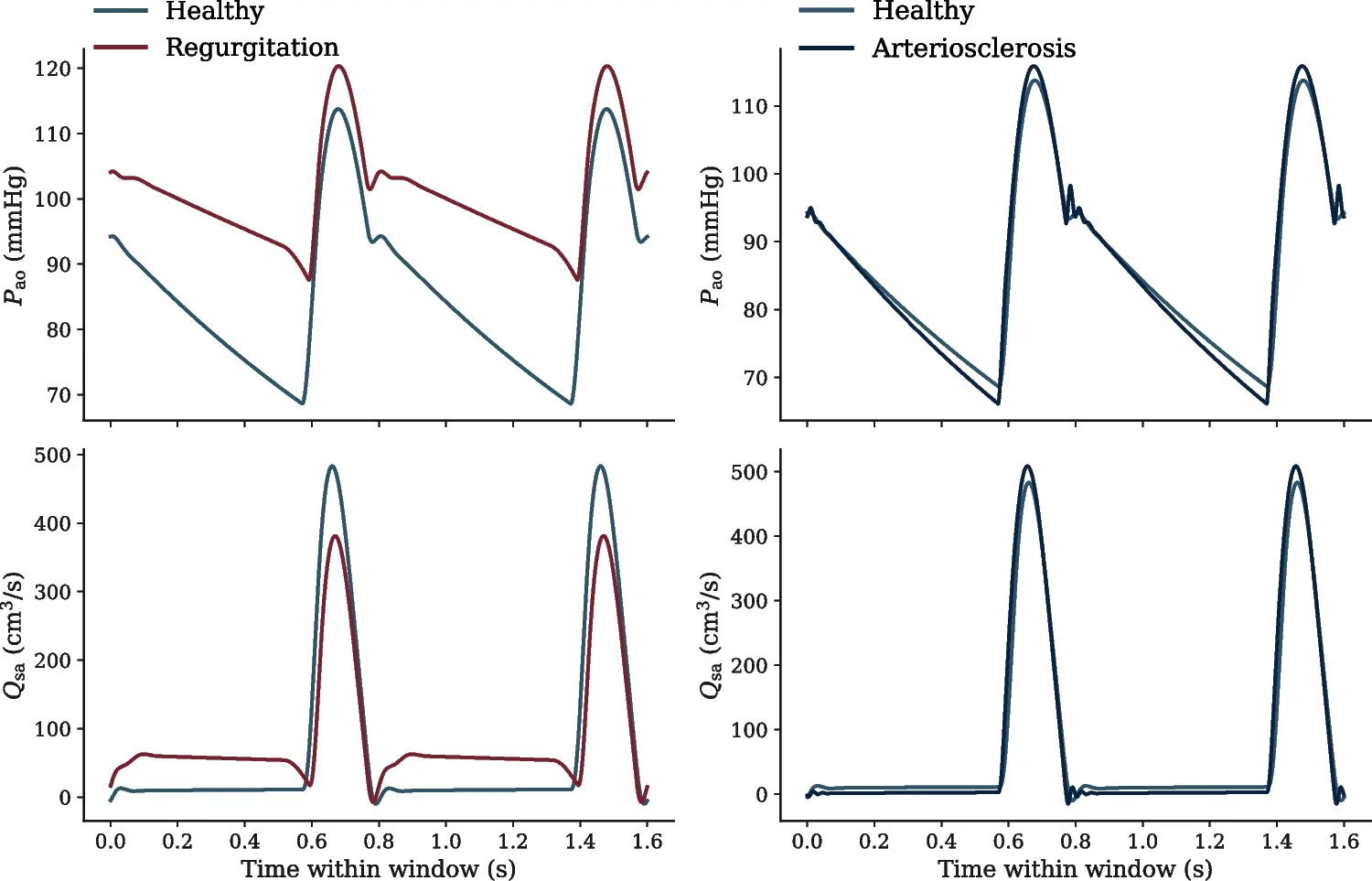
Pathophysiological emulation of aortic regurgitation and arteriosclerosis. Aortic pressure $P_{\textrm{ao}}$ and systemic arterial flow $Q_{\textrm{sa}}$ are compared with the nominal baseline over two steady-state cardiac cycles. Panels (**a**) and (**c**) show the regurgitation scenario, whereas panels (**b**) and (**d**) show the arteriosclerosis scenario, highlighting distinct waveform signatures produced by targeted parameter changes

In the regurgitation scenario, the modified aortic-valve law allows partial retrograde flow during the closed phase. The steady-state response (left column of Fig. 14) shows a widened aortic pressure waveform, reduced systolic flow peaks, and oscillatory flow structures during diastole. The hemodynamic indices shift toward a larger pulse pressure, with $\textrm{PP}=53.8$ mmHg compared with 45.2 mmHg in the nominal baseline, driven mainly by reduced DBP and increased stroke volume ($\textrm{SV}=75.4$ cm$^{3}$). Within the model, this larger stroke volume reflects increased ventricular filling associated with the regurgitant valve pathway, rather than an explicitly regulated compensatory mechanism. These features are qualitatively consistent with clinical descriptions of regurgitant aortic dynamics [43].

#### Arterial stiffening

In the stiffness-dominated scenario, the reduced value of *A* and the increased value of *b* impose a less compliant pressure-dependent aortic element. The steady-state response (right column of Fig. 14) shows a moderate increase in systolic loading, diastolic oscillatory structures in aortic pressure, and a corresponding modulation of systemic arterial flow. These features indicate stronger coupling between proximal stiffness and pulsatile waveform morphology within the lumped network, without implying spatially resolved wave reflection. The hemodynamic indices shift toward elevated SBP and PP ($\textrm{SBP}=115.9$ mmHg, $\textrm{PP}=49.8$ mmHg), while SV and CO remain comparatively preserved. These trends extend the stiffness-sensitivity results of Section 3.2 to a regime combining lower effective compliance with sharper pressure transitions, qualitatively consistent with reported features of advanced arterial stiffening [41, 43].

## Discussion

The Windkessel paradigm provides a mature lumped-parameter framework for macrocirculatory hemodynamics, with a transparent mapping between electrical-analog elements and physiological quantities and a modular architecture in which local changes preserve global consistency. The present model occupies a deliberate position within this landscape: a closed-loop, twelve-state, zero-dimensional formulation embedding a single mechanistically interpretable nonlinearity, the pressure-dependent aortic compliance, within an otherwise classical structure. Spatially distributed phenomena such as pulse-wave reflection and segment-specific elasticity require one- or three-dimensional treatments [9, 44, 45] and lie outside the present scope by construction; in return, the formulation retains the interpretability and computational modesty that make reduced-order models attractive for sensitivity analysis, hypothesis testing, and device-oriented exploration.

A central contribution of the formulation is the pressure-dependent aortic compliance as an explicit nonlinear element within an otherwise classical closed-loop Windkessel, preserving backward compatibility with the linear baseline (Figs. 6 and 7, Table 3) and agreeing with established reference models [14, 16]. Within the exponential law, *b* acts as a compact surrogate for arterial stiffness, aligning with the literature on arterial stiffening, vascular aging, hypertension, and adverse cardiovascular outcomes [29, 46–50]. The compliant regime narrows pulse pressure; the stiffer regime widens it; in both, MAP, SV, and CO remain essentially unchanged, with proximal pulse pressure spanning approximately 36 to 49 mmHg across the sweep while SV and CO stay within narrow ranges (Table 3). The nonlinear capacitor therefore acts as a *controlled gateway*: quiet at nominal physiology, where it reproduces the linear baseline, and progressively expressive away from it, with broader phase portraits and reflection-like structures in the $(x_1, x_2)$ plane (Fig. 8a, b). In this setting, nonlinear effects appear only as the vascular response moves away from the nominal operating range, so the added compliance law gives those changes a direct mechanical interpretation.

Beyond global indices, the closed-loop coupling reproduces two waveform features routinely used in clinical assessment. The reconstructed pressure-volume loops (Fig. 9) exhibit the four canonical phases, with end-systolic and end-diastolic volumes, peak pressures, and stroke volumes within the ranges reported by established closed-loop lumped models [14, 16] and within physiological norms for the resting healthy adult. These loops are not imposed but emerge from the interaction of time-varying elastance, valve dynamics, and the upstream and downstream lumped compartments, providing a non-trivial validation of the cardiac module independent of pressure-waveform comparisons. The dicrotic notch (Fig. 10) is reproduced with a lag of approximately 0.07 s after the systolic peak, consistent with reported physiological values [41], together with the secondary diastolic rise. That a single-node zero-dimensional representation can generate this feature, traditionally invoked as a signature of distributed wave dynamics, accords with prior observations that valve switching and proximal pressure-flow inertance suffice for both even without explicit 1D propagation [27]. Both features survive the nonlinear compliance modification, confirming that the proposed extension does not degrade waveform signatures clinicians routinely inspect.

The closed-loop coupling was probed under four single-parameter challenges (Fig. 11): an afterload increase, a faster heart rate, an increase in left-ventricular contractility, and a reduction of venous return through elevated systemic-venous compliance. The first three reproduce the textbook lumped-parameter responses [14]: hypertensive shift under afterload; elevated mean pressure with reduced per-beat SV offset by higher beat frequency under tachycardia; widened LV pressure-volume loop with elevated SV and SBP under increased contractility. The preload-reduction challenge produced only modest changes in SBP, MAP, SV, and CO. In the closed-loop model used here, where explicit autonomic regulation is not included, the shift of blood volume toward the more compliant venous reservoir is largely accommodated by the periodic dynamics of the system. The combined action of the time-varying ventricular elastance and the systemic inertance limits the response, so the mean operating point does not undergo a sustained displacement. Reproducing the SV and MAP reductions observed clinically during severe orthostatic stress would require explicit volume removal from the systemic compartment or a baroreflex-mediated heart-rate response, neither of which is included here. The same proximal–distal pattern observed in the sensitivity analysis is also present in the challenge scenarios (Fig. 12). In all perturbations, the pressure shift is more pronounced at the proximal site, while the distal response remains attenuated after passing through the lumped systemic network. This behavior supports the interpretation that the nonlinear compliance mainly modulates proximal pulsatility, with part of this effect being filtered before it reaches the peripheral compartment. The pathological emulations of aortic regurgitation and arteriosclerosis (Fig. 14, Table 3) reinforce this picture from a complementary angle: both phenotypes are obtained through localized parameter changes without altering the closed-loop architecture; the arteriosclerosis case in particular highlights distortions inaccessible in a constant-compliance formulation.

Across the sensitivity sweep (Fig. 8a, b), the distal-to-proximal pulse-pressure ratio remains close to 0.63 for *b ∈ [0.010, 0.040]* mmHg$^{-1}$, indicating attenuation of pulsatility from proximal to distal compartment. This contrasts with the central-to-peripheral pulse-pressure amplification reported *in vivo* [9, 41, 44, 45], which arises from wave reflection along a spatially distributed arterial tree and cannot be reproduced by any single-segment 0D representation. The model is therefore complementary to higher-dimensional formulations: it captures the aortic-wall constitutive nonlinearity as a proximal pulsatility regulator without claiming distributed-wave fidelity. A natural extension is a 0D-1D coupling in which the present nonlinear element replaces the proximal lumped capacitance of a 1D arterial tree, preserving the mechanistic interpretability of the constitutive law. The pulmonary bed was also examined because it operates over a much narrower pressure interval, approximately 15 to 28 mmHg. Along this orbit, the factor $e^{-b_{\textrm{pul}}\Delta P}$ changes only slightly, so the induced pulse-pressure modulation remains much smaller than in the systemic circulation, roughly one order of magnitude lower. The systemic response is practically unaffected, with no detectable spillover in Fig. 13. Restricting the nonlinearity to the systemic circulation is therefore a deliberate choice justified by the operating-pressure window of each bed.

The numerical analysis adds a final methodological observation. Both an adaptive explicit Runge-Kutta solver and a stiffly stable implicit method converge to essentially identical steady-state indices across the regimes explored (Table 3); solver choice therefore does not alter the reported physiology, but it reveals the computational character of the regime. As *b* increases and effective compliance collapses, the model develops sharper transients and stronger time-scale separation, manifesting as numerical stiffness. Fixed-step explicit schemes may become fragile or inefficient in this regime, whereas stiffly stable implicit methods remain robust. This stiffness signature works as an operational marker that the system has entered a regime in which pressure-dependent compliance materially reshapes pulsatility while mean loading and pump output remain preserved, and motivates solver-aware pipelines for sensitivity analysis: pathological sweeps should be performed with implicit integrators to avoid conflating numerical artifacts with physiological effects.

Beyond these methodological notes, the model sits within a broader landscape spanning spatial dimensionality and the form of nonlinearity admitted into the arterial-wall constitutive relations. Its spatial complement is the family of one- and three-dimensional arterial-tree models in which pulse-wave propagation, reflection, and segment-specific elasticity can be resolved [9, 43–45], a complementarity articulated in recent reviews of multiscale and reduced-order strategies [51–53]. Fractional-order Windkessel models have also been proposed for aortic impedance and hypertensive hemodynamics [12, 13]; the present integer-order, mechanistically interpretable formulation is a complementary alternative. Two parallel lines generalize the constitutive elements of the Windkessel. The first, to which this work belongs, generalizes the compliance by allowing the elastic-storage term to depend on instantaneous luminal pressure, a line originating with Cappello et al. [21] and developed by Burattini and Natalucci [54]. The second generalizes the resistance by allowing peripheral resistance to depend on flow, pressure, or downstream metabolic demand, capturing autoregulation phenomena [55–57]. Both modify the diastolic decay constant *τ = RC* through mechanistically distinct substrates: peripheral vascular tone at the microcirculatory scale, versus aortic-wall constitutive nonlinearity at the macroscopic vessel-wall scale. A natural future direction is a joint Windkessel containing both $C_n(P)$ and *R*(*P*, *Q*), which would in principle allow their respective contributions to the diastolic decay to be disentangled.

The 0D lumped-parameter formulation, while well suited to the analysis of global hemodynamic indices, carries several structural limitations that identify priority directions for future extensions.

Windkessel-class models cannot reproduce the spatially distributed phenomena of pulse-wave propagation, including the *in vivo* pulse-pressure amplification between central and peripheral sites in healthy young adults. Amplification arises from wave reflection and from the distributed mechanical properties of the arterial tree, neither of which can be represented in a lumped capacitive-resistive-inertial network, which means this limitation is structural rather than numerical. In the sensitivity analysis discussed above, the distal-to-proximal pulse-pressure ratio remained close to 0.63 across *b ∈ [0.010, 0.040]* mmHg$^{-1}$. A hybrid 0D-1D coupling, in which the present nonlinear element replaces the proximal lumped capacitance of a 1D arterial tree, is the natural way to address this directly while preserving the mechanistic interpretability of the constitutive law.

The model treats peripheral resistance as fixed, neglecting neurogenic, myogenic, and metabolic feedback. A joint Windkessel with both nonlinear compliance and dynamically regulated peripheral resistance is the natural follow-up. The disparate time scales of the two nonlinearities (milliseconds-scale constitutive response versus seconds-to-minutes autoregulatory adjustment) mean they act on distinct portions of the hemodynamic response and may, in principle, be separable in clinical data of appropriate temporal resolution. The muted preload-reduction response noted earlier reflects precisely this absence of autonomic feedback.

The model has been validated against canonical hemodynamic indices of the resting healthy adult and against reference closed-loop lumped models [14, 16], but has not been calibrated against individual-subject *in vivo* recordings. Future work directly calibrating *A* and *b* of Eq. (10) against catheter or non-invasive aortic-pressure recordings, possibly together with arterial-stiffness indices such as pulse-wave velocity or augmentation index, would extend the framework’s practical utility. Such subject-specific calibration is the natural bridge between the present mechanistic framework and the data-driven and machine-learning surrogates increasingly prevalent in cardiovascular modeling [58, 59].

The cardiac period $t_c$ is held constant, neglecting heart-rate variability and baroreflex coupling; the ventricular elastance functions $E_L(t)$ and $E_R(t)$ follow a fixed deterministic profile; and the coronary and cerebral circulations are not represented. These extensions lie outside the present scope. A deeper dynamical-systems characterization of the nonlinear regimes identified here, including bifurcation structure, attractor robustness, and stability descriptors, would be a complementary refinement.

Taken together, these analyses support a focused conclusion: introducing pressure-dependent aortic compliance as an explicit nonlinear element in a classical closed-loop Windkessel model increases physiological expressiveness without sacrificing interpretability or computational accessibility. Near the nominal operating point, the nonlinear formulation reproduces the linear baseline. Away from that point, it selectively modulates proximal pulsatility while largely preserving mean loading, stroke volume, and cardiac output. The same mechanism remains compatible with waveform-level markers, including the dicrotic notch and ventricular pressure-volume loops, and responds to physiological and pathological perturbations in patterns consistent with expected hemodynamic behavior. The main structural limitation is intrinsic to the 0D representation: peripheral pulse-pressure amplification cannot be resolved without spatial propagation. This limitation defines constructive extensions toward hybrid 0D-1D formulations, Windkessel architectures combining nonlinear compliance with nonlinear peripheral resistance, and subject-specific calibration against *in vivo* recordings.

## Conclusions

This study introduced a closed-loop, twelve-state, zero-dimensional cardiovascular model in which pressure-dependent aortic compliance, defined by $C_n(P) = A\,e^{-bP}$, is embedded as a single explicit nonlinear element within a classical Windkessel architecture. The formulation preserves the interpretability and computational efficiency of lumped-parameter modeling while extending the framework to stiffness-mediated hemodynamics. Under nominal conditions, the nonlinear model recovers the linear baseline in waveform morphology, phase-space structure, and steady-state indices. Away from that operating point, the compliance law selectively modulates proximal pulsatility while MAP, stroke volume, and cardiac output remain comparatively preserved.

The numerical results support the physiological consistency of the framework at waveform, index, and scenario levels. Increasing aortic stiffness widens proximal pulse pressure and reshapes the pressure-flow limit cycle. The model also preserves ventricular pressure-volume loops, the dicrotic notch, and the diastolic secondary pressure rise. The physiological challenges produce responses in the expected direction, whereas targeted parameter changes generate qualitative signatures consistent with aortic regurgitation and arterial stiffening. At the same time, the central-to-distal analysis confirms a structural limitation of the 0D formulation: spatial pulse-wave propagation and peripheral pulse-pressure amplification require a distributed arterial model.

These findings motivate three extensions: coupling the nonlinear compliance element to a 1D arterial tree, combining $C_n(P)$ with regulated peripheral resistance *R*(*P*, *Q*), and calibrating *A* and *b* against subject-specific pressure recordings and arterial-stiffness indices. In this form, the proposed framework provides a compact, physiologically grounded basis for sensitivity analysis, pathological emulation, and future integration with data-driven cardiovascular modeling.

## References

[1] Quarteroni A, Formaggia L (2004) Mathematical modelling and numerical simulation of the cardiovascular system. Handb Numer Anal 12:3–127. 10.1016/S1570-8659(03)12001-7

[2] Marini JJ, Crooke PS (1993) A general mathematical model for respiratory dynamics relevant to the clinical setting. Am Rev Respir Dis 147(1):14–24. 10.1164/ajrccm/147.1.14 8420408

[3] Albanese A, Cheng L, Ursino M, Chbat NW (2016) An integrated mathematical model of the human cardiopulmonary system: model development. Am J Physiol-Heart Circulat Physiol 310(7):899–921. 10.1152/ajpheart.00230.2014 26683899

[4] Ježek F, Kulhánek T, Kaleckỳ K, Kofránek J (2017) Lumped models of the cardiovascular system of various complexity. Biocybernet Biomed Eng 37(4):666–678. 10.1016/j.bbe.2017.08.001

[5] Liu H, Liang F, Wong J, Fujiwara T, Ye W, Tsubota K-I, Sugawara M (2015) Multi-scale modeling of hemodynamics in the cardiovascular system. Acta Mech Sin 31:446–464. 10.1007/s10409-015-0416-7

[6] Shi Y, Lawford P, Hose R (2011) Review of zero-d and 1-d models of blood flow in the cardiovascular system. Biomed Eng Online 10:33. 10.1186/1475-925X-10-33 PMC3103466 21521508

[7] Ghigo AR, Fullana J-M, Lagrée P-Y (2017) A 2d nonlinear multiring model for blood flow in large elastic arteries. J Comput Phys 350:136–165. 10.1016/j.jcp.2017.08.039

[8] Zhou S, Xu L, Hao L, Xiao H, Yao Y, Qi L, Yao Y (2019) A review on low-dimensional physics-based models of systemic arteries: application to estimation of central aortic pressure. Biomed Eng Online 18:65. 10.1186/s12938-019-0660-3 PMC6446386 30940144

[9] Westerhof N, Lankhaar J-W, Westerhof BE (2009) The arterial windkessel. Med Biol Eng Comput 47(2):131–141. 10.1007/s11517-008-0359-2 18543011

[10] Frasch HF, Kresh JY, Noordergraaf A (1996) Two-port analysis of microcirculation: an extension of Windkessel. Am J Physiol-Heart Circulat Physiol 270(1):376–385. 10.1152/ajpheart.1996.270.1.H376 8769774

[11] Kind T, Faes TJC, Lankhaar J-W, Vonk-Noordegraaf A, Verhaegen M (2010) Estimation of three- and four-element Windkessel parameters using subspace model identification. IEEE Trans Biomed Eng 57(7):1531–1538. 10.1109/TBME.2010.2041351 20172779

[12] Bahloul MA, Laleg-Kirati T (2020) Assessment of fractional-order arterial Windkessel as a model of aortic input impedance in humans. IEEE Open J Eng Med Biol 1:123–132. 10.1109/OJEMB.2020.2988179 PMC8974663 35402942

[13] Bahloul MA, Aboelkassem Y, Laleg-Kirati T (2022) Human hypertension blood flow model using fractional calculus. Front Physiol 13:838593. 10.3389/fphys.2022.838593 PMC8980459 35392372

[14] Korakianitis T, Shi Y (2006) Numerical simulation of cardiovascular dynamics with healthy and diseased heart valves. J Biomech 39(11):1964–1982. 10.1016/j.jbiomech.2005.06.016 16140309

[15] Korakianitis T, Shi Y (2006) Effects of atrial contraction, atrioventricular interaction and heart valve dynamics on human cardiovascular system response. Med Eng Phys 28(7):762–779. 10.1016/j.medengphy.2005.11.005 16376600

[16] Avanzolini G, Barbini P, Cappello A, Cevenini G (1988) Cadcs simulation of the closed-loop cardiovascular system. Int J Biomed Comput 22(1):39–49. 10.1016/0020-7101(88)90006-2 3346090

[17] Ferreira A, Chen S, Simaan MA, Boston JR, Antaki JF (2005) A nonlinear state-space model of a combined cardiovascular system and a rotary pump. In: Proceedings of the 44th ieee conference on decision and control, pp 897–902. 10.1109/CDC.2005.1582271. IEEE

[18] Fogliardi R, Di Donfrancesco M, Burattini R (1996) Comparison of linear and nonlinear formulations of the three-element windkessel model. Am J Physiol-Heart Circulat Physiol 271(6):2661–2668. 10.1152/ajpheart.1996.271.6.H2661 8997329

[19] Cappello A, Gnudi G (1993) Analysis of the arterial pressure-volume curve in the three-element windkessel model. In: Proceedings of computers in cardiology conference pp 385–388. 10.1109/CIC.1993.378423

[20] Li JK-J, Zhu Y (1994) Arterial compliance and its pressure dependence in hypertension and vasodilation. Angiology 45(2):113–117. 10.1177/000331979404500205 8129185

[21] Cappello A, Gnudi G, Lamberti C (1995) Identification of the three-element windkessel model incorporating a pressure-dependent compliance. Ann Biomed Eng 23(2):164–177. 10.1007/BF02368323 7605053

[22] Li JK, Cui T, Drzewiecki GM (1990) A nonlinear model of the arterial system incorporating a pressure-dependent compliance. IEEE Trans Biomed Eng 37(7):673–678. 10.1109/10.55678 2394455

[23] Halaimi A, Chetti B, Laribi B, Labbadlia O (2020) Simulation of a closed loop model equivalent electronic of normal cardiovascular system and valvular aortic stenosis. J Mech Med Biol 20(1):1950074. 10.1142/S021951941950074X

[24] Navrotskaia AA, Anisimov A A (2022) Simulation of blood flow parameters using Frank’s windkessel model of the arterial system. In: 2022 Conference of Russian Young Researchers in Electrical and Electronic Engineering (ElConRus), pp 1549–1552. IEEE. 10.1109/ElConRus54750.2022.9755766

[25] Langewouters GJ, Wesseling KH, Goedhard WJA (1984) The static elastic properties of 45 human thoracic and 20 abdominal aortas in vitro and the parameters of a new model. J Biomech 17(6):425–435. 10.1016/0021-9290(84)90034-4 6480618

[26] Wesseling KH, Jansen JRC, Settels JJ, Schreuder JJ (1993) Computation of aortic flow from pressure in humans using a nonlinear, three-element model. J Appl Physiol 74(5):2566–2573. 10.1152/jappl.1993.74.5.2566 8335593

[27] Stergiopulos N, Westerhof BE, Westerhof N (1999) Total arterial inertance as the fourth element of the Windkessel model. Am J Physiol-Heart Circulat Physiol 276(1):81–88. 10.1152/ajpheart.1999.276.1.H81 9887020

[28] Chemla D, Hébert J, Zamani K, Coirault C, Lecarpentier Y, Fromes Y (1998) Total arterial compliance estimated by stroke volume-to-aortic pulse pressure ratio. Am J Physiol-Heart Circulat Physiol 274(2):500–505. 10.1152/ajpheart.1998.274.2.H500 9486253

[29] Randall OS, Bos GC, Westerhof N (1984) Systemic compliance: Does it play a role in the genesis of essential hypertension? Cardiovasc Res 18(8):455–462. 10.1093/cvr/18.8.455 6467262

[30] Diourté B, Siché J-P, Comparat V, Baguet J-P, Mallion J-M (1999) Study of arterial blood pressure by a Windkessel-type model: Influence of arterial functional properties. Comput Methods Programs Biomed 60(1):11–22. 10.1016/S0169-2607(99)00002-4 10430459

[31] Hairer E, Nørsett SP, Wanner G (1993) Solving Ordinary Differential Equations I: Nonstiff Problems, 2nd edn. Springer Series in Computational Mathematics, vol 8. Springer, Berlin, Heidelberg. 10.1007/978-3-540-78862-1

[32] Dormand JR, Prince PJ (1980) A family of embedded Runge-Kutta formulae. J Comput Appl Math 6(1):19–26. 10.1016/0771-050X(80)90013-3

[33] Butcher JC (2016) Numerical Methods for Ordinary Differential Equations, 3rd edn. John Wiley & Sons, Chichester, UK

[34] Virtanen P, Gommers R, Oliphant TE, Haberland M, Reddy T, Cournapeau D, Burovski E, Peterson P, Weckesser W, Bright J, Walt SJ, Brett M, Wilson J, Millman KJ, Mayorov N, Nelson ARJ, Jones E, Kern R, Larson E, Carey CJ, Polat İ, Feng Y, Moore EW, VanderPlas J, Laxalde D, Perktold J, Cimrman R, Henriksen I, Quintero EA, Harris CR, Archibald AM, Ribeiro AH, Pedregosa F, Mulbregt P, Contributors S (2020) SciPy 1.0: Fundamental algorithms for scientific computing in Python. Nat Methods 17:261–272. 10.1038/s41592-019-0686-2 PMC7056644 32015543

[35] Hairer, E., Wanner, G (1996) Solving Ordinary Differential Equations II: Stiff and Differential-Algebraic Problems, 2nd edn. Springer Series in Computational Mathematics, vol 14. Springer, Berlin, Heidelberg. 10.1007/978-3-662-09947-6

[36] Harris CR, Millman KJ, Walt SJ (2020) Array programming with NumPy. Nature 585:357–362. 10.1038/s41586-020-2649-2 PMC7759461 32939066

[37] Hunter JD (2007) Matplotlib: A 2D graphics environment. Comput Sci Eng 9(3):90–95. 10.1109/MCSE.2007.55

[38] Lynch S (2018) Dynamical Systems with Applications Using Python. Springer, Cham

[39] Lynch S (2023) Python for Scientific Computing and Artificial Intelligence. Chapman and Hall/CRC, Boca Raton, FL

[40] Stergiopulos N, Meister JJ, Westerhof N (1994) Simple and accurate way for estimating total and segmental arterial compliance: The pulse pressure method. Ann Biomed Eng 22(4):392–397. 10.1007/BF02368245 7998684

[41] Nichols WW, O’Rourke MF, Edelman ER, Vlachopoulos C (eds) (2022) McDonald’s Blood Flow in Arteries: Theoretical, Experimental and Clinical Principles, 7th edn. CRC Press, Boca Raton, FL. 10.1201/9781351253765

[42] Magder S (2016) Volume and its relationship to cardiac output and venous return. Crit Care 20(1):271. 10.1186/s13054-016-1438-7 PMC5018186 27613307

[43] Lanzer P (2015) PanVascular Medicine, (2nd edn). Springer, Berlin, Heidelberg. 10.1007/978-3-642-37078-6

[44] Willemet M, Alastruey J (2014) Arterial pressure and flow wave analysis using time-domain 1-d hemodynamics. Ann Biomed Eng 43(1):190–206. 10.1007/s10439-014-1087-4 PMC4286649 25138163

[45] Mynard JP, Smolich JJ (2015) One-dimensional haemodynamic modeling and wave dynamics in the entire adult circulation. Ann Biomed Eng 43(6):1443–1460. 10.1007/s10439-015-1313-8 25832485

[46] Laurent S, Boutouyrie P, Asmar R, Gautier I, Laloux B, Guize L, Ducimetière P, Benetos A (2001) Aortic stiffness is an independent predictor of all-cause and cardiovascular mortality in hypertensive patients. Hypertension 37(5):1236–1241. 10.1161/01.HYP.37.5.1236 11358934

[47] Vlachopoulos C, Aznaouridis K, Stefanadis C (2010) Prediction of cardiovascular events and all-cause mortality with arterial stiffness: A systematic review and meta-analysis. J Am Coll Cardiol 55(13):1318–1327. 10.1016/j.jacc.2009.10.061 20338492

[48] Ben-Shlomo Y, Spears M, Boustred C et al (2014) Aortic pulse wave velocity improves cardiovascular event prediction: An individual participant meta-analysis of prospective observational data from 17,635 subjects. J Am Coll Cardiol 63(7):636–646. 10.1016/j.jacc.2013.09.063 PMC4401072 24239664

[49] Laurent S, Cockcroft J, Van Bortel L, Boutouyrie P, Giannattasio C, Hayoz D, Pannier B, Vlachopoulos C, Wilkinson I, Struijker-Boudier H (2006) Expert consensus document on arterial stiffness: Methodological issues and clinical applications. Eur Heart J 27(21):2588–2605. 10.1093/eurheartj/ehl254 17000623

[50] Mitchell GF, Hwang S-J, Vasan RS, Larson MG, Pencina MJ, Hamburg NM, Vita JA, Levy D, Benjamin EJ (2010) Arterial stiffness and cardiovascular events: The Framingham heart study. Circulation 121(4):505–511. 10.1161/CIRCULATIONAHA.109.886655 PMC2836717 20083680

[51] Scarsoglio S, Ridolfi L (2021) 0D and 1D multiscale computational modeling of coronary circulation: A review. Rev Cardiovasc Med 22(4):1461–1473. 10.31083/j.rcm2204150 34957785

[52] Muskat JC, Rayz VL, Goergen CJ, Babbs CF (2021) Hemodynamic modeling of the circle of Willis reveals unanticipated functions during cardiovascular stress. J Appl Physiol 131(3):1020–1034. 10.1152/japplphysiol.00198.2021 34264126

[53] Shimizu S, Une D, Kawada T, Hayama Y, Kamiya A, Shishido T, Sugimachi M (2018) Lumped parameter model for hemodynamic simulation of congenital heart diseases. J Physiol Sci 68(2):103–111. 10.1007/s12576-017-0585-1 PMC10717555 29270856

[54] Burattini R, Natalucci S (1998) Complex and frequency-dependent compliance of viscoelastic windkessel resolves contradictions in elastic windkessels. Med Eng Phys 20(7):502–514. 10.1016/S1350-4533(98)00055-1 9832026

[55] Lanzarone E, Liani P, Baselli G, Costantino ML (2007) Model of arterial tree and peripheral control for the study of physiological and assisted circulation. Med Eng Phys 29(5):542–555. 10.1016/j.medengphy.2006.08.004 17011809

[56] Aletti F, Lanzarone E, Costantino ML, Baselli G (2009) Simulation study of autoregulation responses of peripheral circulation to systemic pulsatility. Nonlinear Biomed Phys 3:7. 10.1186/1753-4631-3-7 PMC2722629 19630959

[57] Baselli G, Porta A, Pagani M (2006) Coupling arterial windkessel with peripheral vasomotion: Modeling the effects on low-frequency oscillations. IEEE Trans Biomed Eng 53(1):53–64. 10.1109/TBME.2005.859787 16402603

[58] Nataraj C, Hemmati S (2025) Harnessing nonlinear mechanics to transform medical diagnostics. Appl Mech Rev 77:010801. 10.1115/1.4070319. Accepted manuscript PMC12757994 41487433

[59] Varanis M, Hemmati S, Filipus MC, Abreu FLd, Balthazar JM, Nataraj C (2024) An overview on time-frequency effects of ECG signals using synchroextracting transform. In: Lacarbonara, W (ed) Advances in Nonlinear Dynamics, Volume III, pp 591–600. Springer, Cham. 10.1007/978-3-031-50635-2_54

